# Recent Advances in Niobium-Mediated Organic Synthesis: Catalytic Modes, Reactivity, Limitations, and Biomass Valorization

**DOI:** 10.1021/acsomega.6c05715

**Published:** 2026-10-24

**Authors:** Vitor F. Moreno, Maria L. C. D. Tomás, José H. Faleiro, Lucas M. Martins, Giovanny C. dos Santos, Adilson Y. Furuse, Luiz C. da Silva-Filho, Bruno H. S. T. da Silva

**Affiliations:** † Federal University of Uberlândia (UFU), Institute of Chemistry, Uberlândia, Minas Gerais 38400-902, Brazil; ‡ 42466 University of São Paulo (USP), Bauru School of Dentistry, Bauru São Paulo 17012-900, Brazil; § Universidade Federal do Oeste do Pará (UFOPA), Institute of Science and Technology (ICED), Santarém, Pará 68040-255, Brazil; ∥ University of São Paulo (USP), Faculty of Philosophy, Science and Letters at Ribeirão Preto (FFCLRP), Ribeirão Preto, São Paulo 14040-901, Brazil; ⊥ 153994 São Paulo State University (UNESP), School of Sciences, Bauru, São Paulo 17033-360, Brazil

## Abstract

This review critically examines the use of niobium-based compounds in organic synthesis and biomass valorization reported over the past decade. The discussion is organized by catalyst/promoter class (NbCl_5_, Nb_2_O_5_, ammonium niobium oxalate, and modified niobium materials), followed by an analysis of their applications in biomass valorization and a critical assessment of their practical limitations. Using a systematic search strategy, this review establishes explicit quantitative criteria to distinguish genuine catalytic performance from stoichiometric promoter-mediated pathways. Key performance parameters, including catalyst loadings, medium effects, recyclability, and comparative benchmarking against relevant non-niobium alternatives, are systematically assessed. In addition, experimentally demonstrated mechanisms are rigorously distinguished from proposed mechanistic pathways. The analysis reveals that niobium systems are most effective when strong substrate activation is combined with water tolerance and ease of recovery, as demonstrated in Nb_2_O_5_-catalyzed transformations. However, NbCl_5_-mediated reactions frequently rely on superstoichiometric loadings and stringent conditions, severely limiting their practical efficiency. Crucially, a targeted quantitative screening of the literature identifies substantial reporting gaps regarding industrial relevance, continuous processing, leaching and deactivation, and standardized green chemistry metrics (e.g., E-factor). Overall, while heterogeneous niobium materials with tunable Lewis/Brønsted acidity emerge as promising platforms, future progress will require a shift from empirical optimization toward computationally guided catalyst design, standardized sustainability evaluations, and process-oriented validation under scalable conditions.

## Graphical abstract

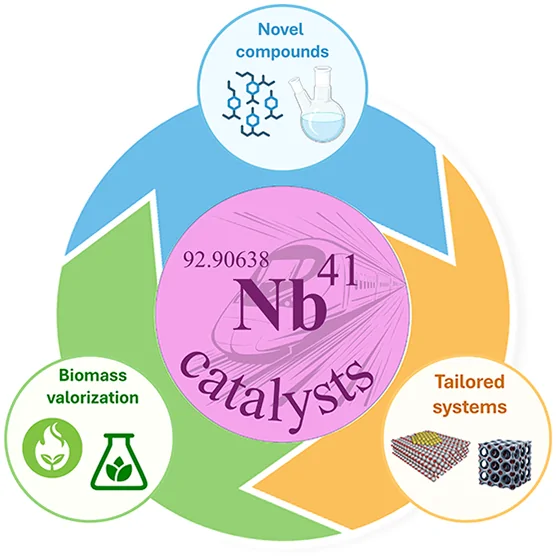

## 1 Introduction

Niobium is a Group 5 transition metal, along with tantalum (Ta) and vanadium (V), with an atomic number of 41 and a relative atomic mass of 92.9064. It does not occur in the metallic state in nature and is typically found in mineral deposits associated with tantalum. Brazil hosts the vast majority of global niobium resources, accounting for approximately 90% of both known global reserves and worldwide market supply.^1–6^

Niobium exhibits high corrosion resistance, good electrical conductivity, and high ductility and malleability, making it well suited for metallurgical and steelmaking applications. Consequently, more than 85% of global niobium production is directed toward the steel and alloy industries.^1–9^ Beyond these large-scale applications, niobium compounds have attracted increasing attention in organic synthesis. Their high oxophilicity, accessible coordination sites, tunable surface acidity, and compatibility with heterogeneous supports enable efficient activation of a broad range of organic substrates. Niobium-based catalysts and promoters can activate carbonyl groups, stabilize oxygenated intermediates, and, in certain systems, display both Lewis and Brønsted acidity, underpinning their versatility in organic transformations.^1–6^

Among the most employed niobium species in organic synthesis are niobium pentachloride (NbCl_5_), niobium pentoxide (Nb_2_O_5_), ammonium niobium oxalate (ANO) and supported or doped niobium-based solids. These systems exhibit distinct reactivity profiles: NbCl_5_ acts as a strong Lewis acid but is highly moisture-sensitive and generally requires anhydrous conditions, whereas Nb_2_O_5_ and supported niobium oxides are thermally stable, reusable, and more aligned with heterogeneous catalytic processes. This diversity of properties plays a central role in catalyst/promoter selection for synthetic applications.^3–5^ More recently, these compounds have played an important role in meeting the growing demand for renewable and cost-effective energy sources as alternatives to fossil fuels, although most of this research is still limited to the laboratory stage, with the potential to scale up to industrial production.^6^

Previous reviews have documented the development of niobium compounds in organic synthesis, including Knoevenagel condensations, Michael additions, Diels–Alder, Mannich, Friedel–Crafts, and Biginelli reactions.^3–5^ In particular, the review published by Arpini et al. in 2015 provided a broad overview of the synthetic applications of niobium compounds reported up to that time and remains an important reference in the field.^5^ Its scope reflected the literature then available and was primarily centered on the types of niobium compounds employed and the organic transformations they promoted. Since its publication, the field has expanded through the development of additional synthetic applications, heterogeneous and structurally modified niobium materials, and the increasing use of niobium-based systems in biomass valorization.

While previous reviews primarily offered descriptive summaries of available transformations, the present work updates the literature (2015–2025) through a reproducible search strategy and introduces a highly critical and comparative framework. Beyond merely cataloging new synthetic applications, in this work the recurring catalytic modes and structure–activity relationships are systematically analyzed, explicitly distinguishing among genuine catalysts, promoter-like reagents, catalyst precursors, or supports. Additionally, niobium loading and reaction conditions are comprehensively assessed, separating experimentally supported mechanisms from proposed mechanistic pathways. Where available data permits, representative niobium-based systems are rigorously benchmarked against non-niobium alternatives. Particular attention is also dedicated to identifying critical reporting gaps related to catalyst recovery, leaching, deactivation, quantitative green chemistry metrics, and process scalability, thereby establishing a clear methodological demarcation from previous literature.

Both homogeneous and heterogeneous systems were considered, including NbCl_5_, Nb_2_O_5_, ammonium niobium oxalate, and modified niobium-containing materials. Their applications in biomass valorization are subsequently examined, followed by a critical assessment of the remaining challenges and research opportunities.

To ensure a reproducible and unbiased assessment, the literature analyzed herein was selected through a systematic search methodology (detailed in Section 2), filtering out nonpeer-reviewed reports to focus strictly on rigorously validated synthetic protocols

## 2 Literature Search Strategy and Selection Criteria

This critical review employed a systematic search strategy, comprising an advanced search executed covering the last full decade (2015 to 2025) across two major electronic databases: Web of Science (WoS) and Scopus. These platforms were selected due to their comprehensive coverage of high-impact, peer-reviewed scientific literature globally recognized within chemistry and materials science.

The literature search was based on key concepts relevant to the scope of this review, using terms such as “niobium”, “niobium pentachloride”, “niobium oxide”, “ammonium niobium oxalate”, “organic synthesis”, “biomass valorization”, and “catalysis”. These keywords were combined using Boolean operators (AND, OR) and adapted to the syntax of each database.

The selection of studies followed predefined inclusion and exclusion criteria. Eligible publications comprised peer-reviewed research articles and critical reviews written in English that addressed the mechanistic aspects, catalytic behavior, and synthetic applications of niobium-mediated transformations. Publications lacking peer review, including conference proceedings, conference papers, books, book chapters, and other documents without sufficient experimental characterization or mechanistic insight, were not considered. Industrial patents were also omitted. Although they provide valuable information on technological applications, patents frequently lack the peer-reviewed validation, mechanistic evidence, and detailed characterization necessary for establishing the structure–activity relationships that constitute the primary focus of this review.

The screening and selection process is illustrated in the flowchart (Figure 1). From an initial pool of 436 records identified across the two databases, 227 duplicate or nonrelevant records were removed, leaving 209 abstracts that underwent screening for scope alignment. Following full-text assessment, 117 articles were omitted due to a lack of focus on organic synthesis, insufficient experimental data, or a lack of mechanistic depth, resulting in the final selection of 91 key publications that form the basis of this critical review.

**Figure 1:**
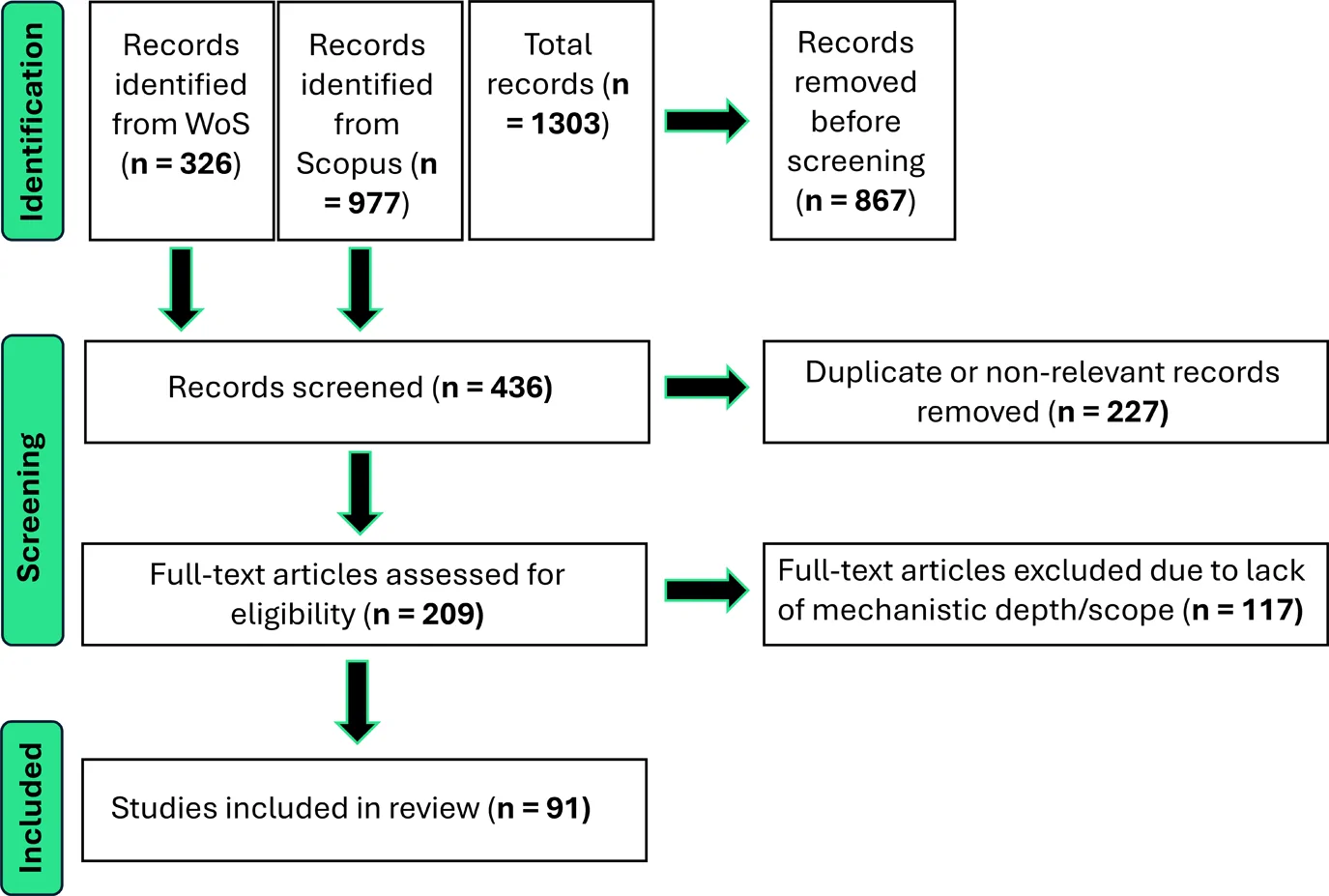
Flow diagram detailing the literature search, screening, and selection strategy used in this review.

### 2.1 Catalytic Modes and Structure–Activity Relationships in Niobium Catalysts

Moving beyond simple yield comparisons, a meaningful assessment of niobium-based systems requires clear criteria to distinguish genuine catalysis from stoichiometric promotion. In this review, based on the reactivity profiles observed in the literature, we establish a practical demarcation: systems operating well below 50 mol % loading are evaluated as catalytic, whereas those requiring 50 mol % or higher (e.g., 0.5–1.0 equiv) are strictly classified as promoter-mediated processes. This distinction is vital, as the frequent reporting of superstoichiometric loadings fundamentally alters the green chemistry footprint and scalability of the transformation, regardless of the isolated yield. Alongside these operational metrics, we critically evaluate systems based on reaction conditions, recyclability, and the requirement for inert atmospheres or specific additives.

Alongside these operational metrics, the fundamental reactivity of niobium can be systematized into recurring activation modes. We categorize the most relevant mechanisms into (i) Lewis acid activation of oxygen- and nitrogen-containing groups,^3–6^ (ii) combined Lewis/Brønsted acid catalysis in heterogeneous oxides, and (iii) cooperative effects arising from surface architecture and metal–support interactions.^12–15^ Figure 2 presents a unifying conceptual overview of these catalytic regimes and their typical characteristics.

**Figure 2:**
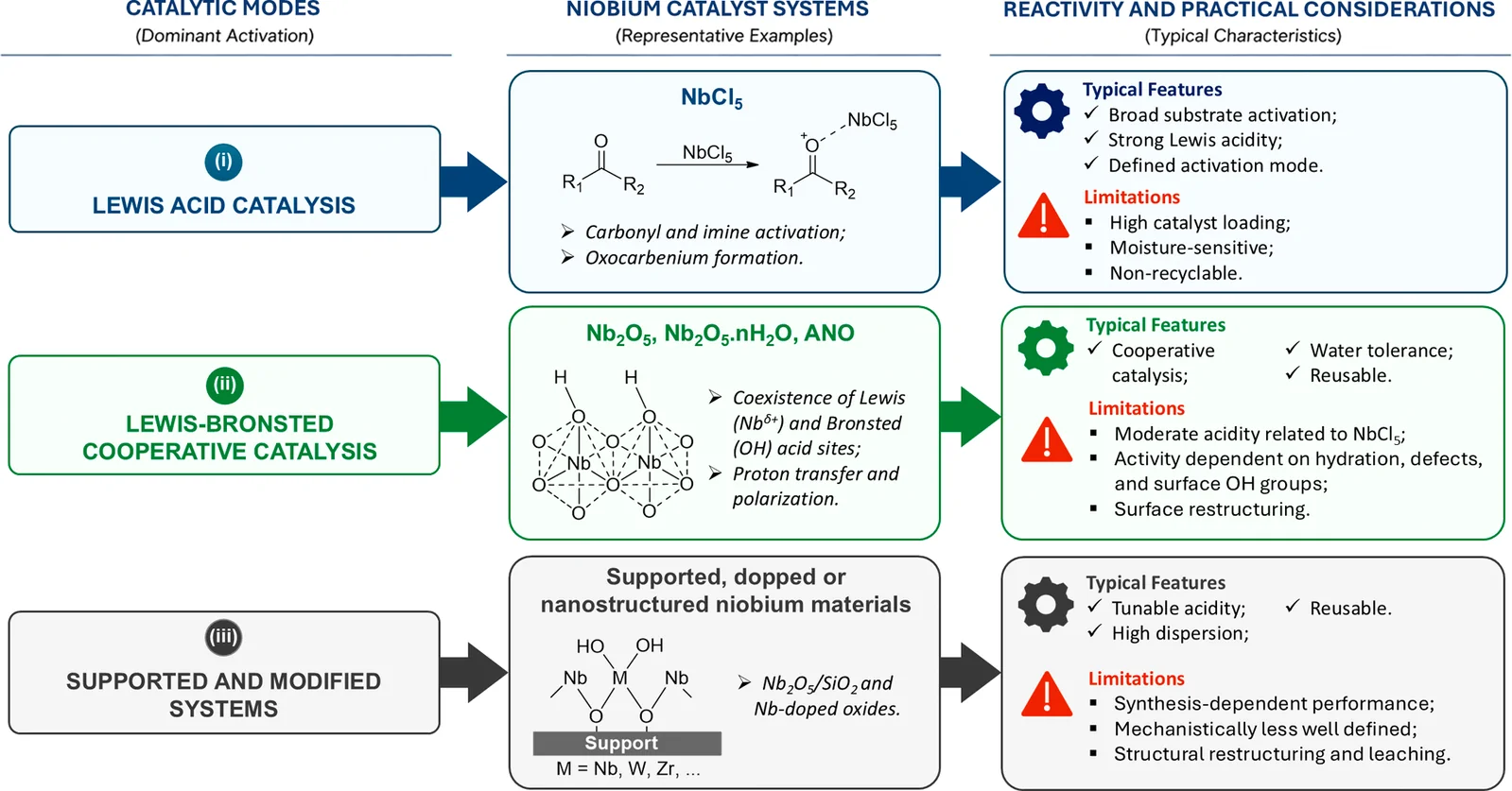
Conceptual framework of catalytic modes in niobium-based systems.

The framework depicted in Figure 2 highlights that niobium-based catalysts/promoters operate through recurring activation modes governed by their structural properties. Molecular systems such as NbCl_5_ remain the most widely encountered niobium reagents in organic synthesis, primarily due to their strong Lewis acidity and broad ability to activate carbonyl, imine, and other heteroatom-containing functional groups.^3–5^ Mechanistically, the strong oxophilicity and high Lewis acidity of Nb­(V) promote coordination to oxygen- or nitrogen-containing functional groups, increasing bond polarization and lowering the activation barrier for nucleophilic attack. Consequently, many transformations proceed through activated carbonyl species, iminium or oxocarbenium intermediates, followed by electrophilic bond-forming events. These pathways support their broad application in condensation reactions, cycloadditions, Friedel–Crafts transformations, and multicomponent reactions.^3–6^ While high yields and selectivities are frequently reported, these systems often require relatively high loadings (25–100 mol %), strictly anhydrous conditions, inert atmospheres, and chlorinated solvents.^3–5^ However, while NbCl_5_ is an undeniably potent Lewis acid, its reliance on stringent and environmentally taxing conditions often limits its practical and industrial viability.

In contrast, niobium oxides and related materials operate through the cooperative action of Lewis and Brønsted acid sites within extended solid-state frameworks.^13–15^ Lewis acid sites associated with coordinatively unsaturated Nb centers, together with Brønsted acid sites often generated by surface hydroxyl groups, promote substrate activation by simultaneously polarizing reactive functional groups and facilitating proton-transfer events. This synergistic behavior is particularly advantageous in reactions involving oxygenated substrates and aqueous media, making these materials well suited for transformations such as dehydration, esterification, transesterification, and photocatalytic processes.^14–17^ Hydrated niobium oxide (Nb_2_O_5_·nH_2_O), crystalline Nb_2_O_5_, ANO, and niobium phosphate exemplify this behavior, offering active sites that remain functional under aqueous conditions. In general, these materials sacrifice intrinsic acidity for improved stability, water tolerance, and recyclability. Their performance is strongly governed by surface properties, including hydration state and defect density.^13–15^

Supported, doped, and nanostructured niobium materials further extend the capabilities of bulk oxides by enabling precise control over surface acidity and site distribution, often improving catalyst dispersion and reusability. However, these advantages are accompanied by a stronger dependence on synthesis conditions, as well as uncertainties regarding the precise nature of the active sites and potential leaching processes. Nonetheless, such systems have been successfully applied across a range of transformations, including transesterification, dehydration, biomass conversion, and multicomponent reactions.^12–15^ Together, these distinctions highlight the central role of catalyst structure in dictating activation pathways and practical performance. This provides a basis for rationalizing reactivity trends and identifying the specific conditions under which niobium-based systems offer meaningful synthetic advantages.

Overall, these observations establish a clear structure–activity relationship in niobium-based catalysts: molecular Nb­(V) Lewis acids generally provide higher intrinsic activity but often require harsher reaction conditions, whereas heterogeneous niobium oxides and modified materials trade part of this intrinsic reactivity for enhanced stability, recyclability, and operational simplicity.

## 3 Niobium-Catalyzed Organic Reactions

### 3.1 Niobium Pentachloride

A systemic limitation in the current literature regarding NbCl_5_-mediated synthesis is the near-exclusive reliance on isolated yields for performance benchmarking. Quantitative kinetic parameters, such as Turnover Number (TON) and Turnover Frequency (TOF), are rarely reported for these homogeneous protocols. Consequently, our benchmarking relies on comparative temporal efficiency (yield per hour) and direct contrasts with non-niobium Lewis acids evaluated under analogous conditions.

Niobium pentachloride (NbCl_5_) is a well-established Lewis acid in organic synthesis, yet its chemistry has continued to evolve over the past decade, expanding its range of reactivity and applications. This evolution reinforces its role as a versatile reagent, although practical limitations remain.

Recent synthetic protocols heavily exploit NbCl_5_ to drive the assembly of quinoline derivatives via multicomponent and tandem processes, highlighting its pronounced effectiveness as a Lewis acid in cyclizations involving anilines, aldehydes, and alkynes (Schemes 1–4). Based on product distribution and standard reactivity patterns, the authors postulated a two-stage mechanistic pathway. In this proposed sequence, NbCl5 initially coordinates to the carbonyl oxygen, increasing the electrophilicity of the carbonyl group and rapidly promoting nucleophilic attack by the aniline to form a Schiff base intermediate. Subsequently, the niobium species coordinates with the alkyne, activating it as a dienophile to react with the previously formed imine (acting as the diene component). This sequence culminates in a slower Diels–Alder cycloaddition, which drives the closure of the quinoline ring. Substrates bearing electron-withdrawing substituents, particularly nitro groups, exhibit enhanced reactivity and afford yields of up to 98%.

**Scheme 1:**
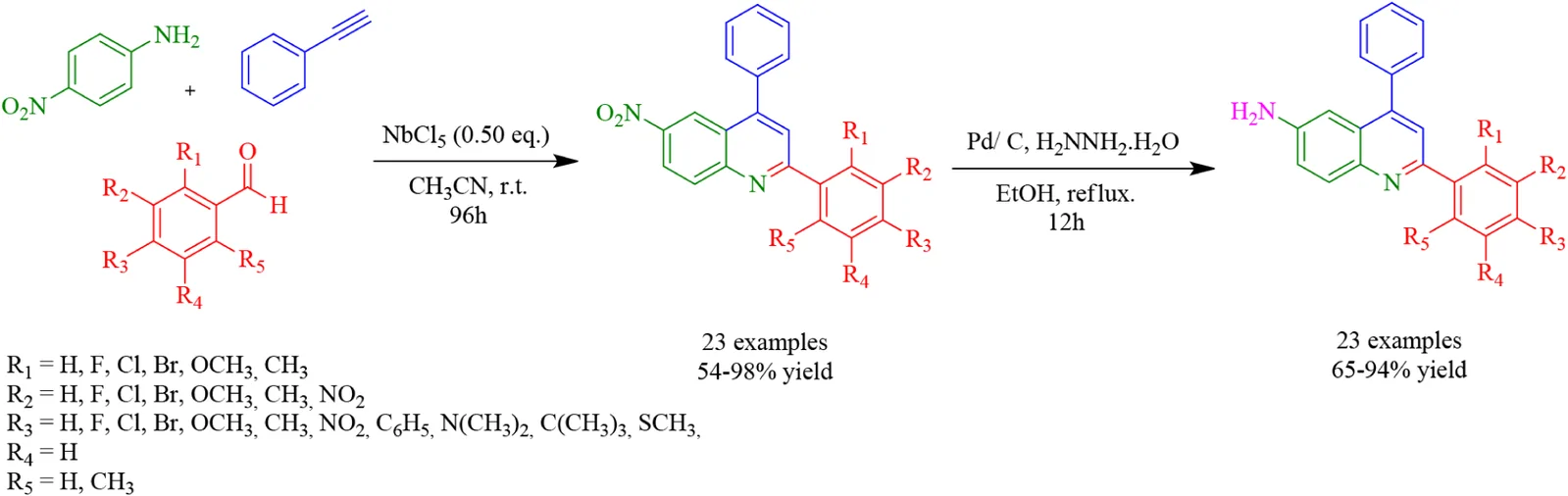
Synthesis of Nitroquinoline Promoted by NbCl_5_

**Scheme 2:**
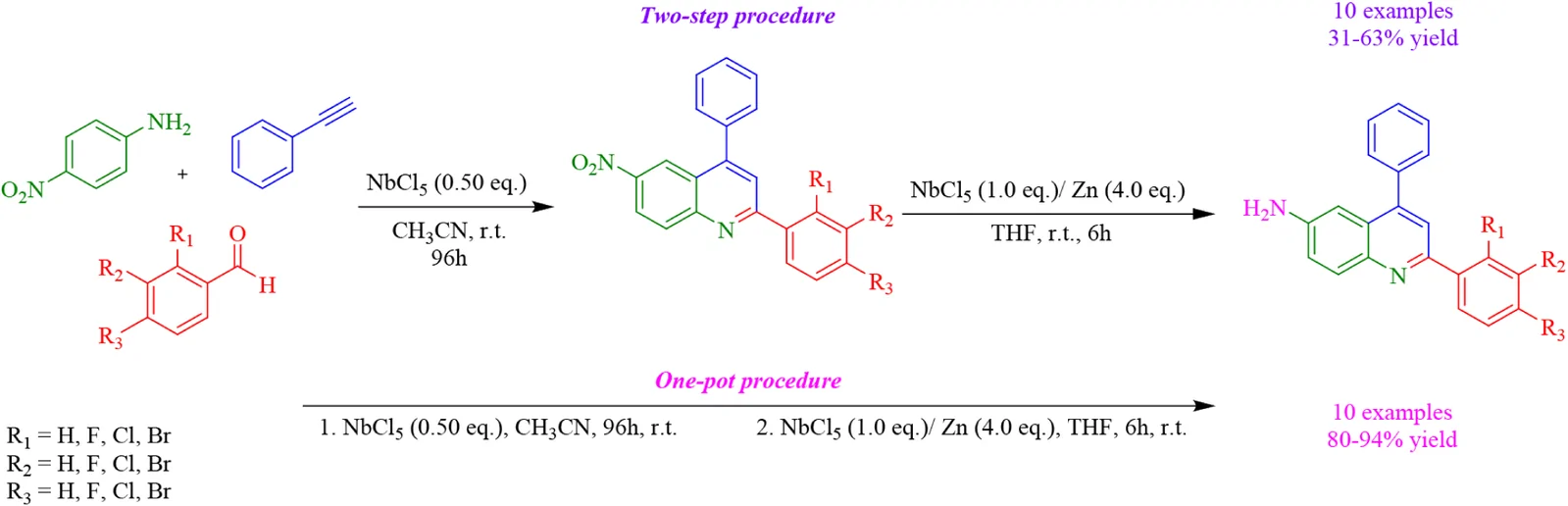
Synthesis of Halogenated Aminoquinolines Promoted by NbCl_5_ and NbCl_5_/Zn Complex

**Scheme 3:**
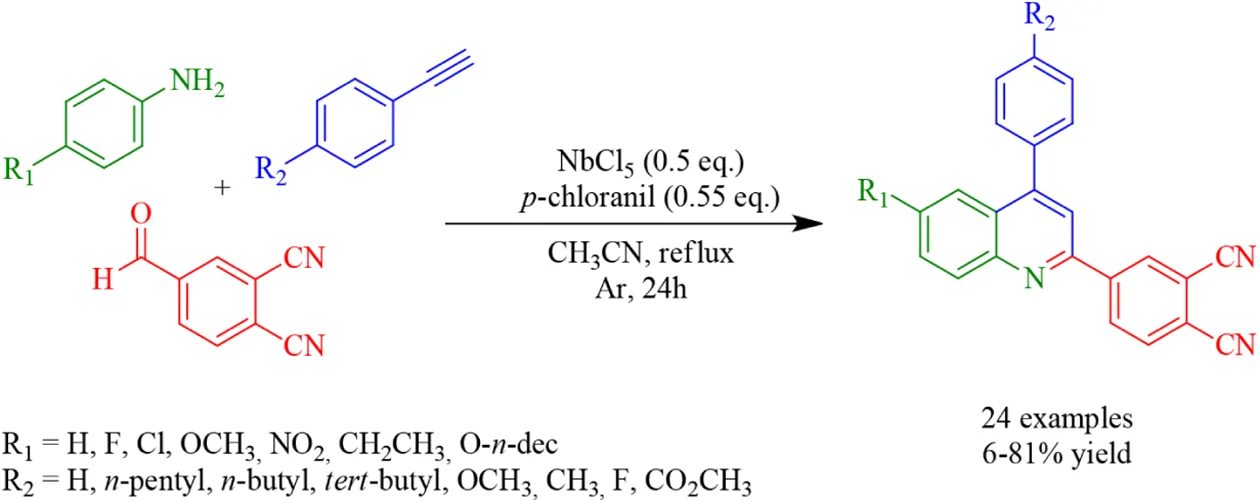
Synthesis of Phthalonitrile–Quinoline Promoted by NbCl_5_

**Scheme 4:**
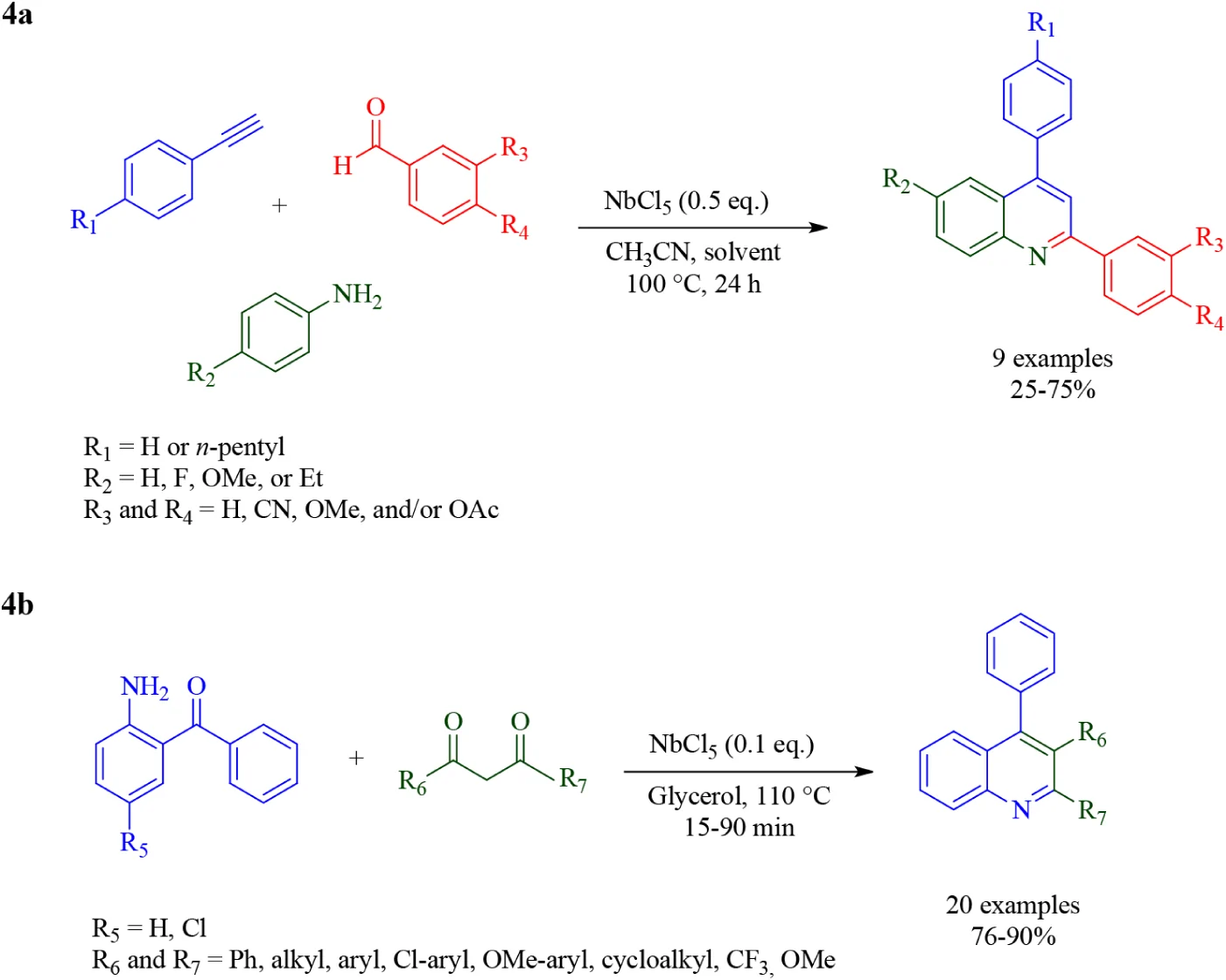
NbCl_5_-Promoted Synthesis of Quinolines: (a) Multicomponent Reaction and (b) Reaction in Glycerol

The authors also compared the performance of NbCl_5_ with several Lewis acids previously reported for the synthesis of 2,4-diphenylquinoline, including FeCl_3_, Yb­(Pfb)_3_, AgNTf_2_/HOTf, InCl_3_, AlCl_3_, and ZnCl_2_. Although NbCl_5_ did not afford the highest isolated yields, its performance was competitive with several established Lewis acids and markedly superior to conventional catalysts such as AlCl_3_ and ZnCl_2_, which produced only trace amounts of product under their optimized conditions. Catalysts such as FeCl_3_ (93%), AgNTf_2_/HOTf (90%), InCl_3_ (90%), and Yb­(Pfb)_3_ (88%) afforded higher yields, however, these comparisons should be interpreted with caution because the reported reactions were performed under substantially different conditions, including distinct solvents (e.g., acetonitrile, toluene, and 1,2-dichloroethane), temperatures (35 °C to reflux), and reaction times (12–288 h). These results indicate that NbCl_5_ offers a balanced compromise between activity and operational simplicity rather than the highest isolated yield.

Furthermore, the resulting nitroquinolines can be further reduced to their corresponding amino derivatives (e.g., using hydrazine/Pd–C), significantly expanding their functional scope (Scheme 1).^9^ These compounds have been successfully investigated as photoinitiators, chemosensing, and optoelectronic applications.^18–22^

Beyond classical Lewis acid activation, NbCl_5_ can also operate in redox-assisted transformations. In the presence of metallic zinc, low-valent niobium species enable single-electron transfer (SET) pathways, promoting the selective reduction of preformed nitroquinolines to amino derivatives in moderate yields (31–63%) while avoiding the dehalogenation typically observed with strong reducing agents such as hydrazine/Pd–C (Scheme 2).^23^ In contrast, a one-pot approach starting from anilines, benzaldehydes, and alkynes integrates quinoline formation and reduction, affording higher yields (80–94%). This comparison underscores the strong influence of reaction design: the one-pot approach operates under milder conditions with shorter reaction times while avoiding hazardous reagents, but the need for near-stoichiometric NbCl_5_ limits its applicability.

Similarly, the combination of NbCl_5_ with oxidants such as *p*-chloranil promotes the aromatization of dihydroquinoline intermediates in multicomponent systems (Scheme 3).^24^ These reactions provide access to phthalonitrile-quinoline derivatives, which serve as precursors to functional materials such as zinc phthalocyanines with promising photophysical properties.^24^ Reported yields vary widely (6–81%) under relatively standard conditions, indicating a strong dependence on substrate structure. The prolonged reaction times (up to 24 h) and, crucially, the use of 0.5 equiv of NbCl_5_ call into question the classification of this methodology as genuinely catalytic. Such high catalyst loadings are more consistent with promoter-like behavior than with efficient catalysis, representing a critical limitation that remains unaddressed in the original report.

Despite the broad scope and structural diversity accessible through these strategies, many of these transformations rely on relatively high catalyst loadings, long reaction times, or additional reagents, raising concerns regarding efficiency and sustainability.^25^ However, recent studies demonstrate that reaction design plays a critical role in addressing these limitations. As illustrated in Scheme 4, conventional quinoline synthesis conditions (Scheme 4a) typically require high NbCl_5_ loadings, whereas the use of alternative media such as glycerol (Scheme 4b) enables faster reactions and significantly reduced catalyst loadings, down to 0.1 equiv, while maintaining high yields (76–90%).^26^ These results highlight practical strategies to enhance the performance of NbCl_5_-based systems.

Several of these nitro and amino-substituted quinoline derivatives have been investigated across a range of applications, including type II photoinitiators for photopolymerization of dimethacrylate monomers used in dental resin-based composites (conversions up to 70%),^18,19^ chemosensors for metallic cations and pH,^20^ as well as semiconducting components of thin films with potential use in LEDs and p–n junctions.^21,22^ They also have potential application as solvatochromic probes, with demonstrated interactions with albumin and lysozyme, enabling the detection of protein denaturation and amyloid aggregation,^27^ as well as for discriminating the surface charge of micelles.^28^

Beyond quinoline synthesis, NbCl_5_ has been widely applied in multicomponent reactions for the construction of diverse heterocyclic frameworks, including pyrrolopyrroles, xanthenediones, chromenes, phloroglucinol, coumarin, and xanthene derivatives (Schemes 5–11).^29–37^ These transformations typically rely on Lewis acid activation of carbonyl compounds, followed by sequential condensation and cyclization steps, often resembling Knoevenagel–Michael reaction sequences.^29–32^

**Scheme 5:**
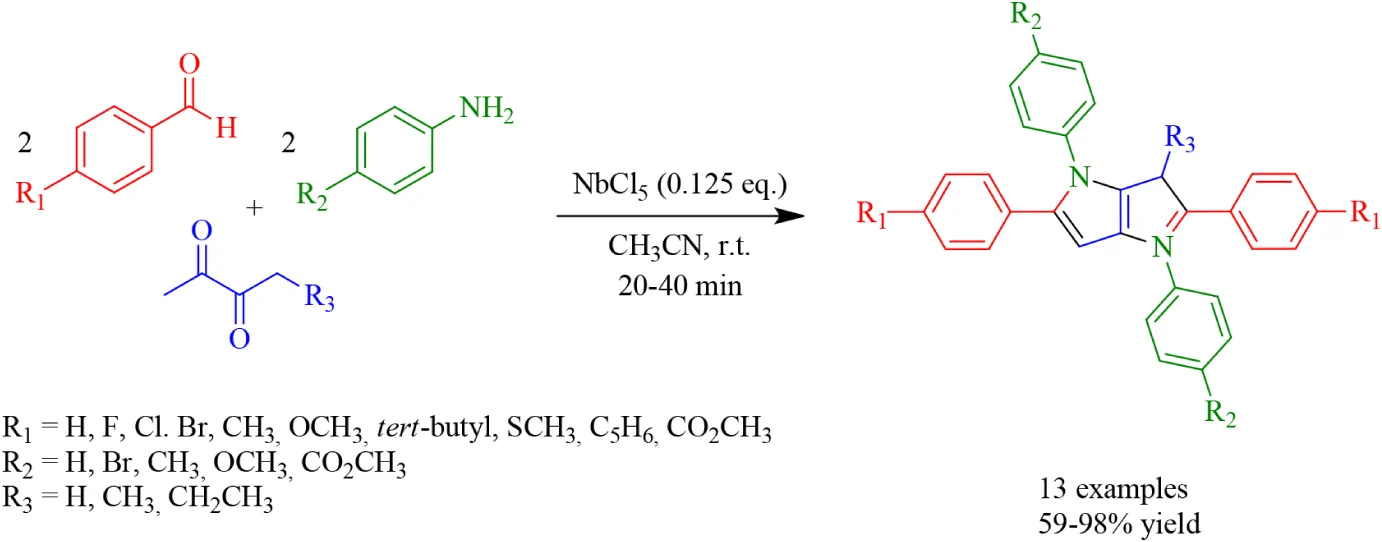
Synthesis of Tetraaryl-1,4-Dihydropyrrolo­[3,2-*b*]­Pyrroles Catalyzed by NbCl_5_

Representative examples include the synthesis of tetraaryl-1,4-dihydropyrrolo­[3,2-*b*]­pyrroles from benzaldehydes, anilines, and α-diketones (Scheme 5), which proceeds efficiently at room temperature using 0.125 equiv of NbCl_5_, affording yields up to 98% within 40 min.^29^ The transformation exhibits pronounced substrate sensitivity; notably, productive outcomes dictate the use of specific α-diketones, particularly 2,3-butanedione, likely due to steric constraints in key condensation steps. Moreover, the pronounced oxophilicity of NbCl_5_ enabled the synthesis of specific derivatives with improved performance compared with Brønsted acid catalysts, including AcOH and TsOH, which required 210 min of reaction time and afforded only 7–34% yields of the desired products under the reported conditions. Electronic structure calculations further indicate competing mechanistic pathways that can be modulated by substrate selection. The resulting compounds exhibit useful optical properties, enabling their application as sensitizing dyes in DSSCs and as fluorescent probes.^30,31^

In a related approach, NbCl_5_ promotes the formation of xanthenedione and phloroglucinol derivatives through one-pot condensations involving cyclic diketones and aldehydes, or cyclic ketones, phenols, and aldehydes, respectively (Schemes 6 and 7).^32,33^ These methodologies deliver target scaffolds in yields ranging from 54% to 87% under reflux conditions, requiring 0.25 equiv of the niobium catalyst, although reaction times can vary significantly (2–24 h). While rigorous operando studies are lacking, the authors proposed a common activation mode involving carbonyl coordination and stabilization of key intermediates along Knoevenagel–Michael-type pathways. Scale-up studies indicate good reproducibility, suggesting synthetic robustness despite the relatively high catalyst loading.^32,33^

**Scheme 6:**
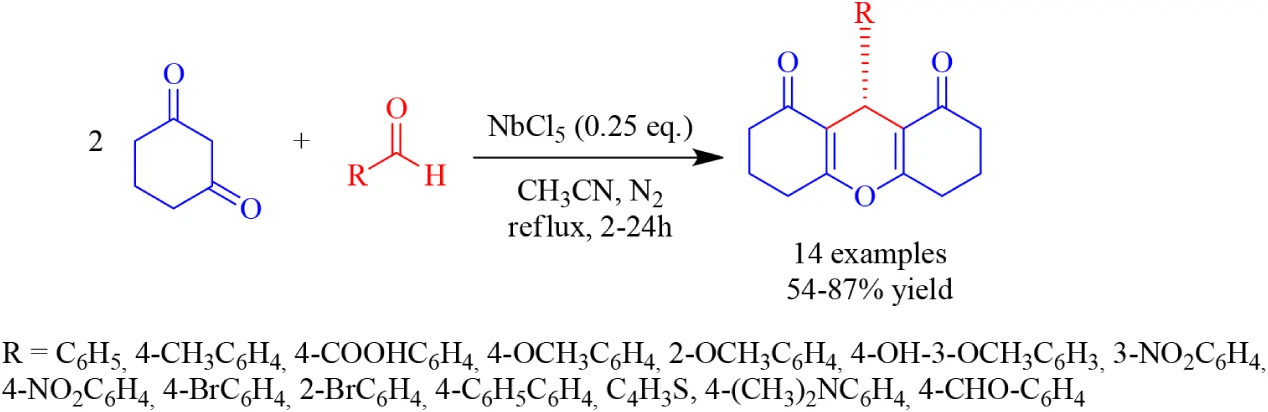
Synthesis of Xanthenedione Derivatives Catalyzed by NbCl_5_

**Scheme 7:**
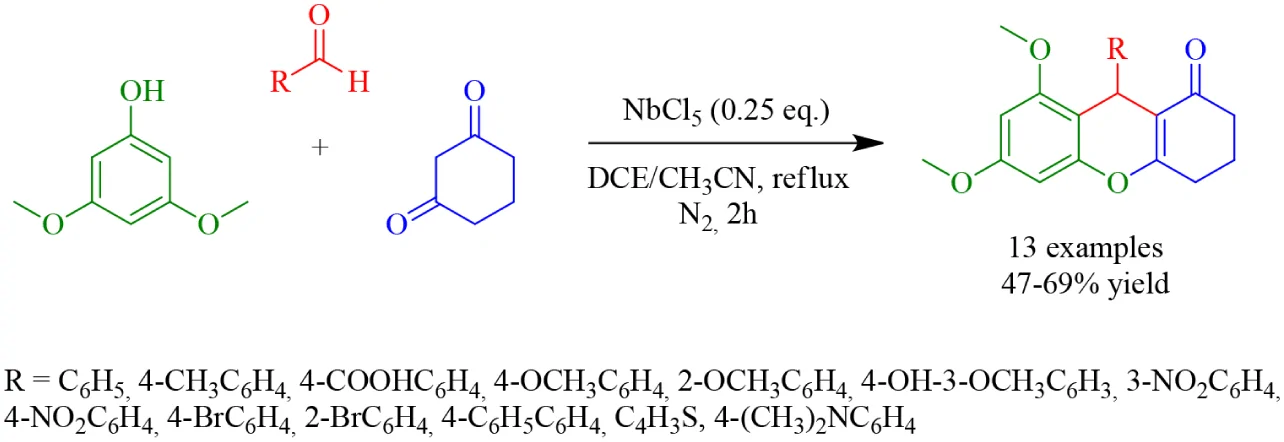
Synthesis of Methoxylated Phloroglucinol Derivatives Catalyzed by NbCl_5_

Extension of this methodology to coumarin-based systems further illustrates the generality of this approach. Multicomponent reactions involving 4-hydroxycoumarin and 4-aminocoumarin promote the formation of chromene and chromenopyridine derivatives, respectively (Schemes 8 and 9), generally in moderate to good yields (38–75%).^34,35^ However, high niobium loadings (up to 1.0 equiv) and extended reaction times are often required, suggesting a promoter-like role of niobium species in these transformations.

**Scheme 8:**
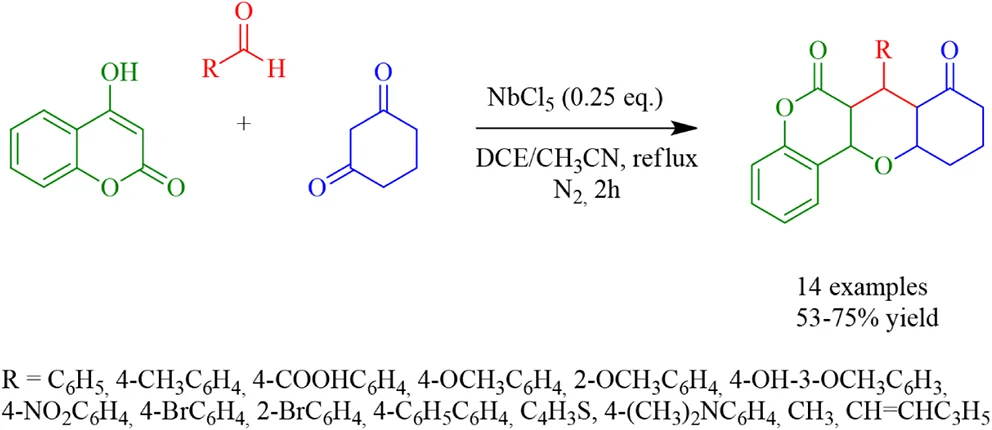
Synthesis of Chromeno­[4,3-*b*]­Chromene Derivatives Catalyzed by NbCl_5_

**Scheme 9:**
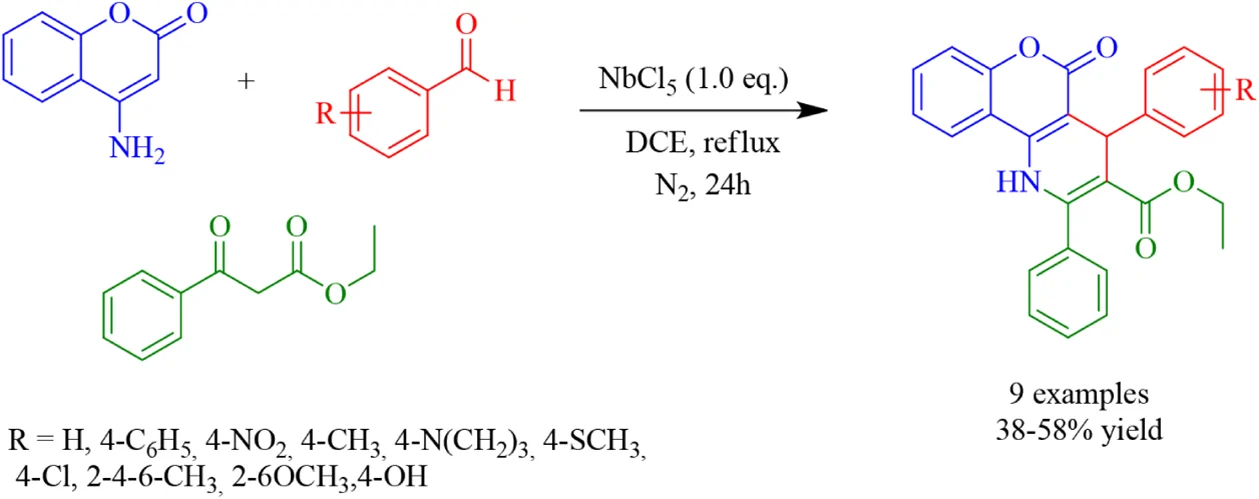
Synthesis of Chromeno­[4,3-*b*]­Pyridines Promoted by NbCl_5_

Within this context, related multicomponent reactions involving phenols, β-ketoesters, and aldehydes afford 4*H*-chromene derivatives via analogous NbCl_5_-mediated pathways (Scheme 10).^36^ Comparative studies with other Lewis acids, such as FeCl_3_ (17% yield) and AlCl_3_ (no reaction), under same reaction conditions and catalyst loading, highlight the higher efficiency of NbCl_5_, which afforded yields of up to 91%. Despite the mild temperatures, the necessity for a full equivalent of NbCl_5_ and prolonged reaction times (up to 72 h) suggests promoter-like behavior rather than genuine catalytic activity.

**Scheme 10:**
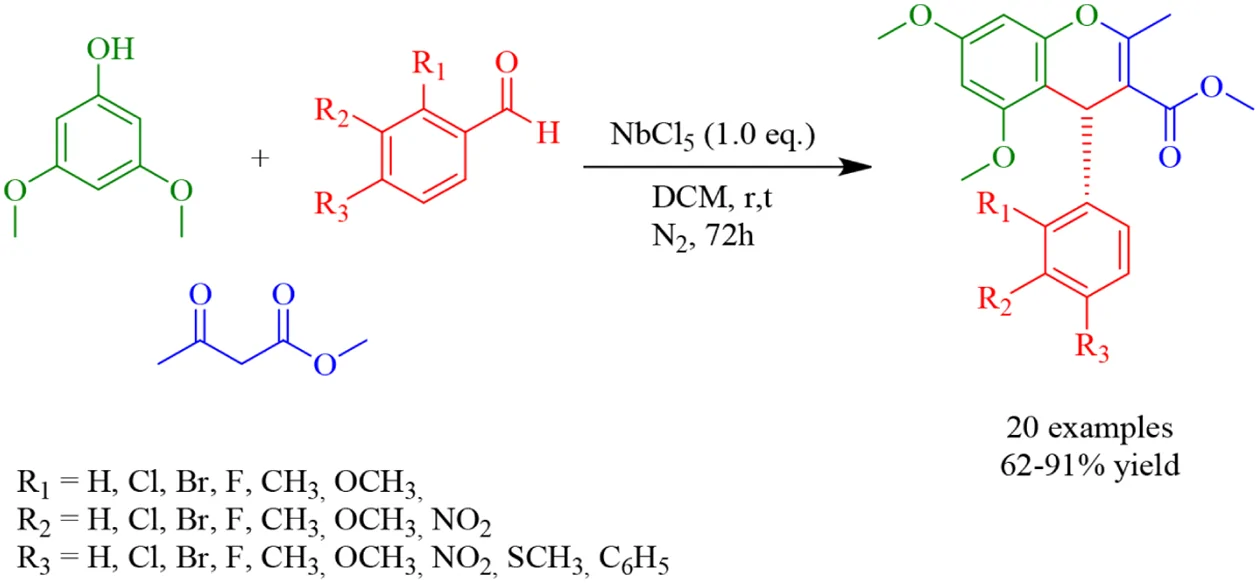
Synthesis of 4*H*-Chromene Derivatives Promoted by NbCl_5_

In a more complex multicomponent scenario (Scheme 11), stoichiometric amounts of NbCl_5_ act as a powerful directing agent, enabling divergent reactivity in the condensation of β-naphthol, dimethyl malonate, and aldehydes. Depending on the aldehyde’s electronic properties, the system exhibits remarkable chemo- and stereoselectivity, strongly favoring *trans*-diequatorial coumarins (41–93%) over xanthenes (1–38%) in the presence of electron-donating groups.^37^ While the structural complexity achieved is impressive, the reliance on such high niobium loadings underscores the absence of a genuine catalytic cycle.

**Scheme 11:**
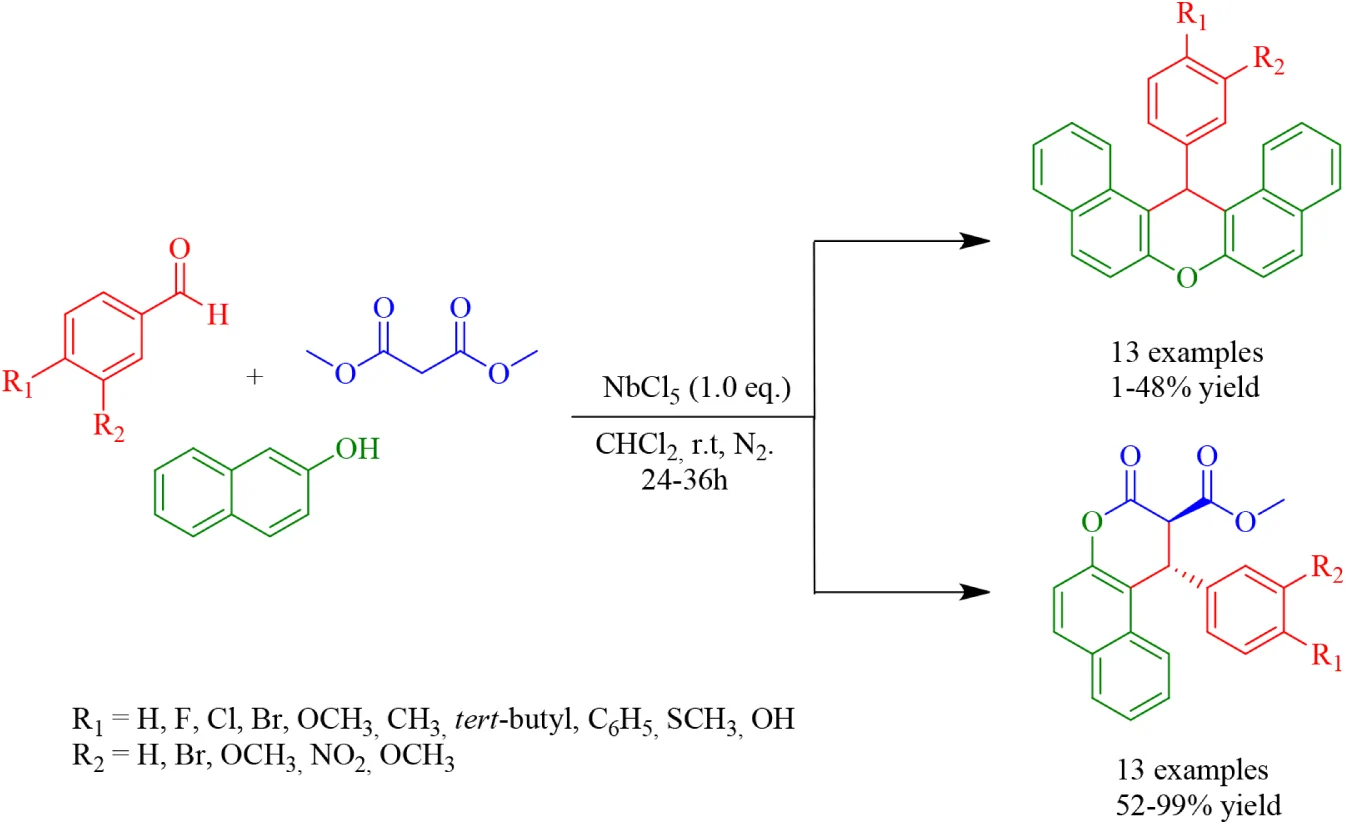
Synthesis of Aryl-3,4-Dihydro-Benzo­[*f*]­Coumarins and 14-Aryl-14H-Dibenzo­[*a, j*]­Xanthenes in the Presence of NbCl_5_

Still in the context of multicomponent condensations, NbCl_5_ promotes the formation of 4H-pyran derivatives from methyl acetoacetate and benzaldehydes (Scheme 12), affording high yields (up to 91%) under mild temperatures and promoter-like conditions.^38^

**Scheme 12:**
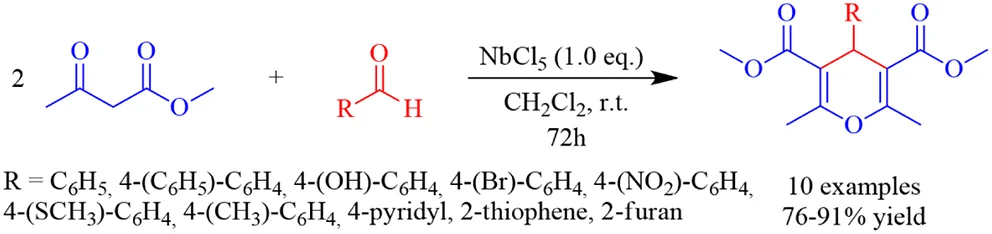
Synthesis of 4*H*-Pyran Derivatives Promoted by NbCl_5_

Beyond condensation-based multicomponent reactions, NbCl_5_ has also been applied across a broader range of transformations, including cycloadditions and electrophilic aromatic substitutions (Schemes 13–16).^38–43^

**Scheme 13:**
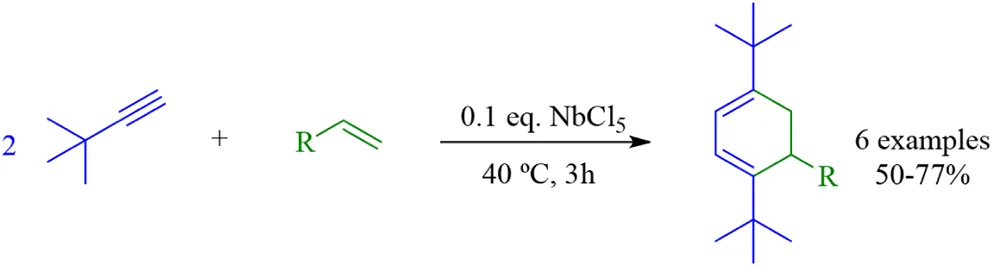
[2 + 2 + 2] Cycloaddition Catalyzed by NbCl_5_

**Scheme 14:**
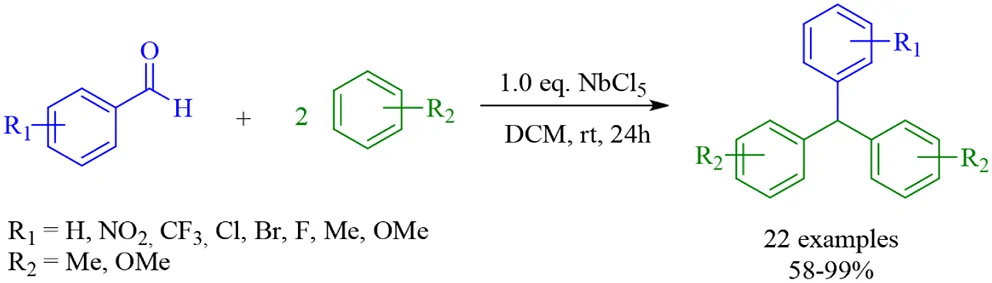
Synthesis of Triarylmethanes Promoted by NbCl_5_

**Scheme 15:**
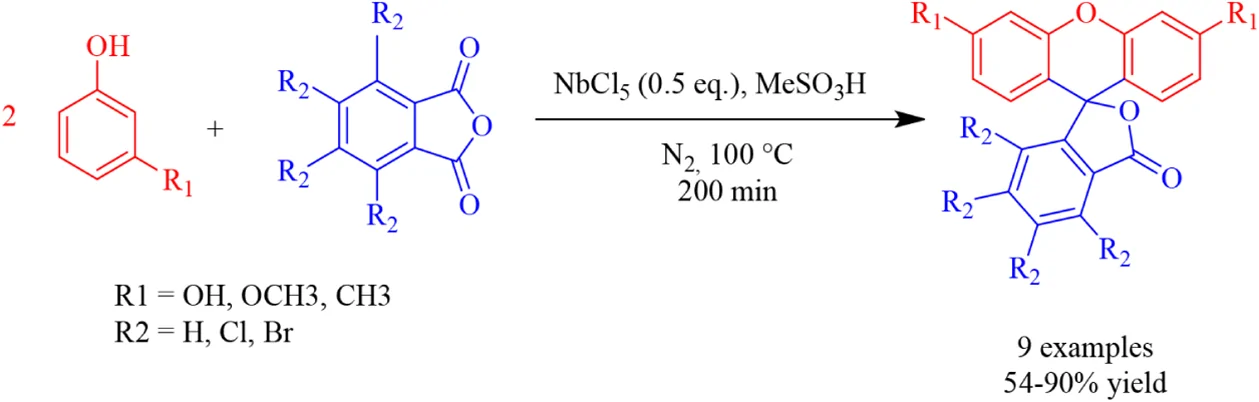
Synthesis of Fluorescein Derivatives in the Presence of NbCl_5_

**Scheme 16:**
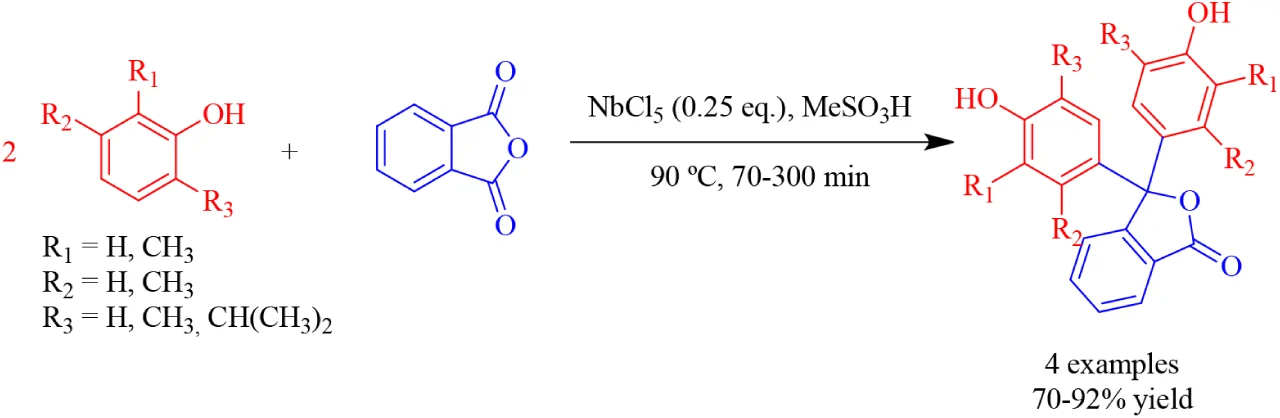
Synthesis of Phthalein Derivatives in the Presence of NbCl_5_

More efficient catalytic behavior is observed in [2 + 2 + 2] cycloaddition reactions (Scheme 13), which have been reported to proceed with high chemo- and regioselectivity without the need for low-valent niobium precursors or external reducing agents. Cyclohexadiene derivatives are obtained in good to excellent yields (up to 94%) using low catalyst loadings and short reaction times. Comparative analyses indicate that NbCl_5_ significantly outperforms other common Lewis acids, such as FeCl_3_, AlCl_3_, and ZrCl_4_, which demonstrate limited efficacy (<32%) under identical reaction conditions. This example highlights that NbCl_5_ can achieve genuine catalytic turnover when the reaction pathway is well aligned with its Lewis acidic character.

NbCl_5_ also exhibits consistent performance in Friedel–Crafts-type transformations, encompassing both alkylation and acylation manifolds (Schemes 14–16).^40–43^ In multicomponent reactions between aldehydes and arenes (Scheme 14), NbCl_5_ affords triarylmethanes (58–99%) under relatively mild conditions.^40^ Similarly, electrophilic acylation/condensation of phthalic anhydrides with phenols affords fluorescein and phthalein derivatives (Schemes 15 and 16) via sequential aromatic substitution and cyclization steps.^41–43^ While these systems generally provide good to high yields (54–92%), their performance depends strongly on the reaction medium and catalyst loading: fluorescein synthesis typically requires higher NbCl_5_ loadings (0.5 equiv) and strongly acidic conditions, whereas phthalein formation proceeds with somewhat lower loadings 0.25 equiv).^41–43^ Specifically, in the synthesis of fluorescein derivatives, the authors benchmarked the NbCl_5_/MeSO_3_H system against several literature-reported catalytic systems, including H_3_PO_4_ and ZnCl_2_ under microwave irradiation, as well as Fe­(HSO_4_)_3_ under conventional heating. Notably, MeSO_3_H alone promoted the formation of the desired products with similar yields however, a reaction time 60-fold longer was required compared with the NbCl_5_/MeSO_3_H system. It is also worth noting that higher yields were only achieved under ultrasound-assisted conditions using H_3_PO_4_ and ZnCl_2_, affording 97% and 90% yields, respectively. The analysis demonstrates that NbCl_5_ provides an efficient catalytic platform, delivering results comparable to other Lewis acids while maintaining shorter reaction times and competitive yields. Notably, the combination of MeSO_3_H and NbCl_5_ significantly accelerates the transformation.

A unifying mechanistic pattern emerges across quinoline, chromene, pyran, and xanthene syntheses, as well as multicomponent, cycloaddition and Friedel–Crafts transformations: NbCl_5_ is particularly effective when the rate-limiting step involves Lewis acid activation of carbonyl or imine functionalities, thereby promoting subsequent nucleophilic addition events. In such multicomponent reactions, the metal often provides useful chemoselectivity and accommodates a broad substrate scope that can challenge weaker Lewis acids. However, a recurring trade-off emerges: the most substrate-specific or forcing conditions frequently coincide with higher niobium loadings, characteristic of a promoter-like reaction.^9,23–28^ A comparative overview of representative NbCl_5_-mediated transformations, including reaction conditions, benchmarking against other Lewis and Brønsted acids, and reaction outcomes, is presented in Table 1.

**Table 1:** Comparative Summary of Representative NbCl_5_-Mediated Transformations, Organized by Reaction Family

Reaction Type	Catalyst	Loading (equiv)	Time (h)	Yield (%)	Conditions	Key Descriptor
Quinoline MCR^9,25^	NbCl_5_	0.5	24–96	98	Anhydrous CH_3_CN and reflux	High yields long reaction times; high catalyst loading.
	FeCl_3_	0.1	4–12	25–74	Toluene and reflux	Lower yields short reaction times; catalytic conditions.
Pyrrolo-pyrrole MCR^29^	NbCl_5_	0.25	0.7	Up to 98	Anhydrous CH_3_CN and no heating	Short reaction times; high yields substrate-sensitive.
	TsOH	0.1	3.5	Up to 49	AcOH and heating	Low yields acidic conditions.
Chromene synthesis^36,38^	NbCl_5_	1.0	72	Up to 91	DCM and rt	High yields stoichiometric catalyst loading; long reaction times.
	AlCl_3_ or FeCl_3_	1.0	72	Up to 17	DCM and rt	No reaction or low yield.
Friedel–Crafts (triarylmethane)^40^	NbCl_5_	1.0	24	Up to 99	DCM and rt	High catalyst loading; long reaction times; stoichiometric catalyst loading.
Friedel–Crafts reactions^41^	NbCl_5_	0.25	12–24	Up to 90	Anhydrous and inert medium; Acidity medium and heating.	High yields high catalyst loading; long reaction times; harsh conditions.
	-	-	40	Up to 74	Acidity medium and heating.	No catalyst; moderate yields with long reaction times.
Cyclo-additions [2 + 2 + 2][Bibr ref39]	NbCl_5_	0.1	3	Up to 77	Neat and mild heating.	High yields and short reaction times.
	ZrCl_4_, FeCl_3_ or AlCl_3_	0.1	3	No reaction observed.	-	-

Overall, the examples discussed herein highlight the versatility of NbCl_5_ as a Lewis acid for promoting a wide range of organic transformations. Its effectiveness is particularly evident in reactions where substrate activation and controlled electrophilic reactivity are crucial; however, its practical applicability is often limited by operational requirements, including heating, moisture-sensitive conditions, and the use of stoichiometric or near-stoichiometric amounts in some reactions, suggesting promoter-like behavior. Therefore, although NbCl_5_ represents a valuable tool in synthetic chemistry, its advantages must be considered in the context of substrate structure, reaction design, and comparison with alternative catalytic systems.

Furthermore, it should be noted that the studies discussed in this section generally focus on the synthetic applicability of NbCl_5_, emphasizing substrate scope, product diversity, yields, and reaction conditions. Consequently, parameters commonly used for evaluating catalytic efficiency, such as green chemistry metrics (e.g., E-factor), kinetic parameters (e.g., TON and TOF), and scale-up feasibility, are rarely reported in these studies, limiting a comprehensive assessment of the catalyst from an industrial and process-oriented perspective.

### 3.2 Niobium Oxide

In contrast to NbCl_5_, niobium oxides are most compelling when robustness, water tolerance, and catalyst recoverability are central to performance. Their reactivity relies less on strong, homogeneous Lewis acidity and more on accessible surface sites, hydroxyl-assisted activation, and heterogeneous operation. As a result, the comparison shifts from isolated yields to overall process behavior, where Nb_2_O_5_ often outperforms simple oxides such as Al_2_O_3_ or TiO_2_, particularly in transformations involving oxygenated substrates or basic byproducts.^44–46^ In this context, Nb_2_O_5_ has been widely explored as a heterogeneous catalyst, either in its bulk form or as a modified material, enabling a range of carbonyl-activation-driven transformations. Representative examples include multicomponent condensations, amidation reactions, hydrolysis processes, and cyclizations, which generally proceed via activation of carbonyl groups at Nb^5+^ surface sites followed by nucleophilic addition and bond-forming steps.^44–49^

In multicomponent condensations, Nb_2_O_5_ efficiently drives the assembly of heterocyclic frameworks under solvent-free conditions. For instance, it mediates the synthesis of rhodamine B-based dyes, delivering 79–90% yields within 1 h, with the added advantage of catalyst recovery (Scheme 17). The resulting compounds exhibit pronounced solvatochromic behavior, attributed to lactone ring opening in polar media, highlighting their applicability in sensing and optoelectronic devices.^44^ However, the high niobium loading compromises the authors’ assertion regarding the catalytic nature of this oxide, suggesting instead a promoter-like behavior.

**Scheme 17:**
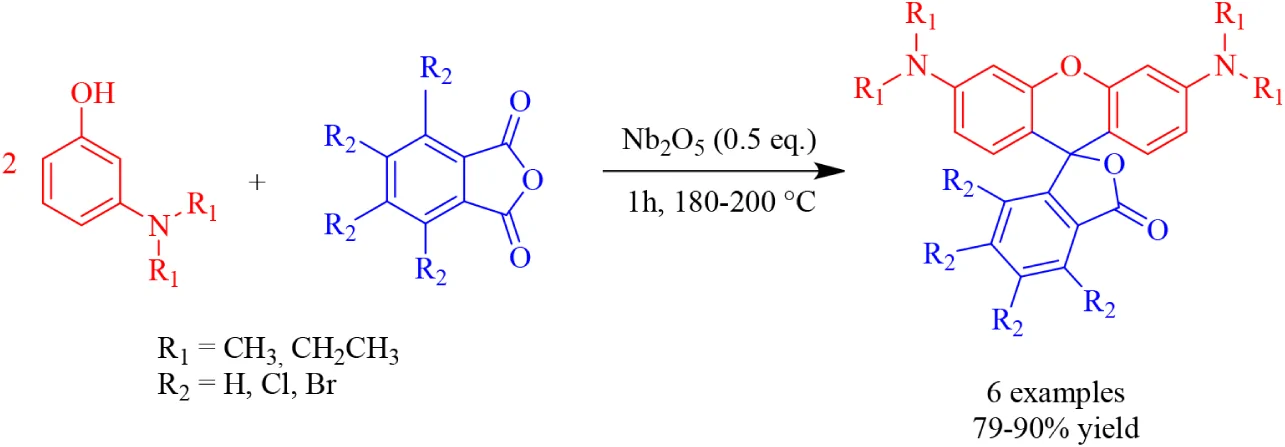
Synthesis of Rhodamine B Derivatives using Nb_2_O_5_

Further expanding this heterogeneous reactivity, Biginelli reactions catalyzed by thermally treated Nb_2_O_5_ (Nb_2_O_5_/T) afford dihydropyrimidinones in excellent yields (up to 94%) under solvent-free conditions at 130 °C, with reaction times below 1.5 h and with catalyst recyclability (Scheme 18). Comparative performance screening against homogeneous H_2_SO_4_ (28%) and alternative niobium-based catalysts, including thermally treated and untreated niobic acid (50–75%) and untreated Nb_2_O_5_ (70%), highlights the superior performance of Nb_2_O_5_/T, where the synergy between thermal and chemical treatments optimizes surface acidity and enhances catalytic efficiency. Notably, the catalyst can be reused for at least three cycles without significant loss of activity.^45^

**Scheme 18:**
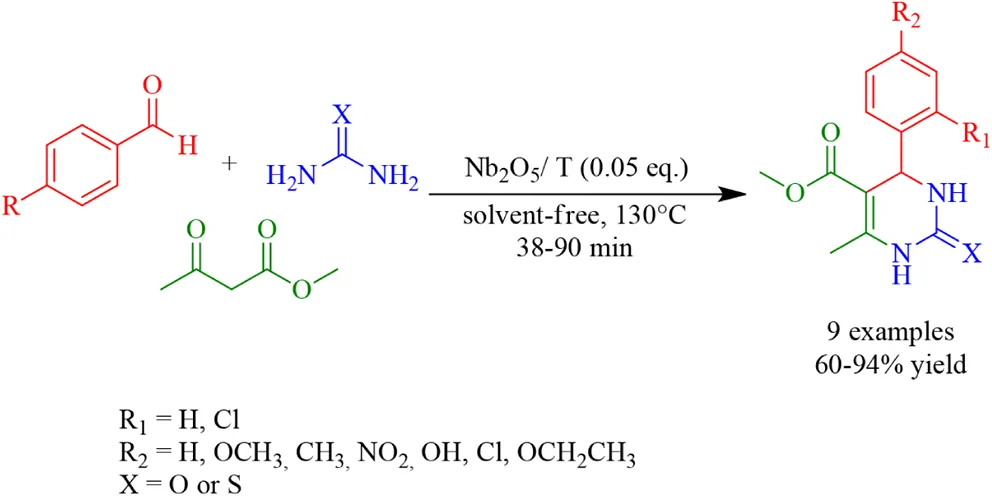
Synthesis of Dihydropyrimidinones Catalyzed by Nb_2_O_5_ /T

These multicomponent reactions highlight the efficiency of Nb_2_O_5_ in solvent-free systems, affording high yields within short reaction times; however, the rhodamine derivatives synthesis requires relatively high catalyst/promoter loadings despite enabling its recovery, whereas the Biginelli reaction proceeds under truly catalytic conditions (as low as 0.05 equiv) with comparable performance. In both cases, the elevated temperatures (130–200 °C) are consistent with solvent-free operation above the melting point of the reactants rather than reflecting intrinsic limitations of the process.

Besides multicomponent reactions, Nb_2_O_5_ exhibits remarkable performance in C–N bond-formation. Direct amidation of carboxylic acids and esters with amines proceeds under heating, delivering amides in yields up to 98% across a broad substrate scope (Scheme 19). Notably, Nb_2_O_5_ significantly outperformed conventional metal oxides such as Al_2_O_3_, TiO_2_, ZnO, and ZrO_2_, which afforded considerably lower yields under similar conditions. Among the Lewis acids evaluated, ZrCl_4_ provided the closest performance to Nb_2_O_5_, affording a 71% yield, whereas Nb_2_O_5_ afforded 99% under the same conditions. This enhanced activity stems from its dual capability: robust activation of the carbonyl bond coupled with pronounced tolerance toward basic species and aqueous byproducts. The weaker adsorption of both amines and water on the Nb_2_O_5_ surface minimized catalyst deactivation and facilitates the C–N bond formation step, which proceeds with a lower energy barrier.^46–48^

**Scheme 19:**
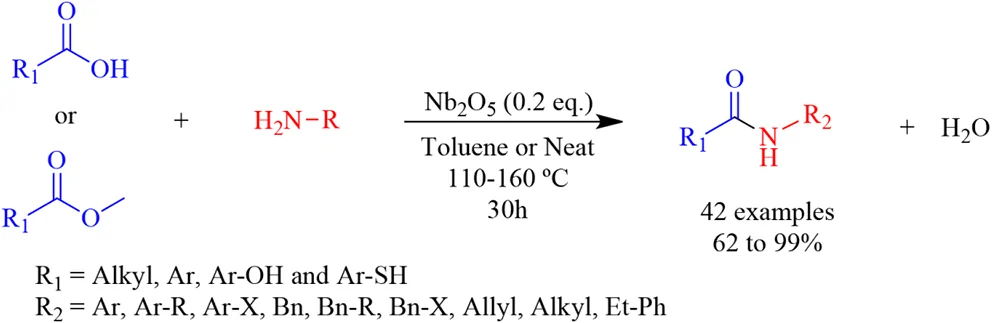
Direct Amidation of Carboxylic Acids and Amines Catalyzed by Nb_2_O_5_

Similar behavior is observed in the synthesis of cyclic imides from anhydrides and amines, where high yields (55–98%) are obtained under comparable conditions (Scheme 20).^47–49^ However, the practical viability of these methodologies is limited by the requirement for elevated temperatures (up to 160 °C) and prolonged reaction times of 30 h. Similar to the direct amidation of carboxylic acids, Nb_2_O_5_ displayed enhanced catalytic performance compared with other metal oxides, including titanium-, tin-, tantalum-, and zinc-based oxides. The closest result was obtained with ZrO_2_, which afforded a 65% yield, whereas Nb_2_O_5_ provided 90% under identical conditions. Despite the use of moderate Nb_2_O_5_ loadings, the catalyst can be recovered and reused, and, when combined with solvent-free conditions, this approach represents a potentially more sustainable alternative compared to conventional methodologies that rely on expensive or toxic catalysts/solvents.^47–49^

**Scheme 20:**
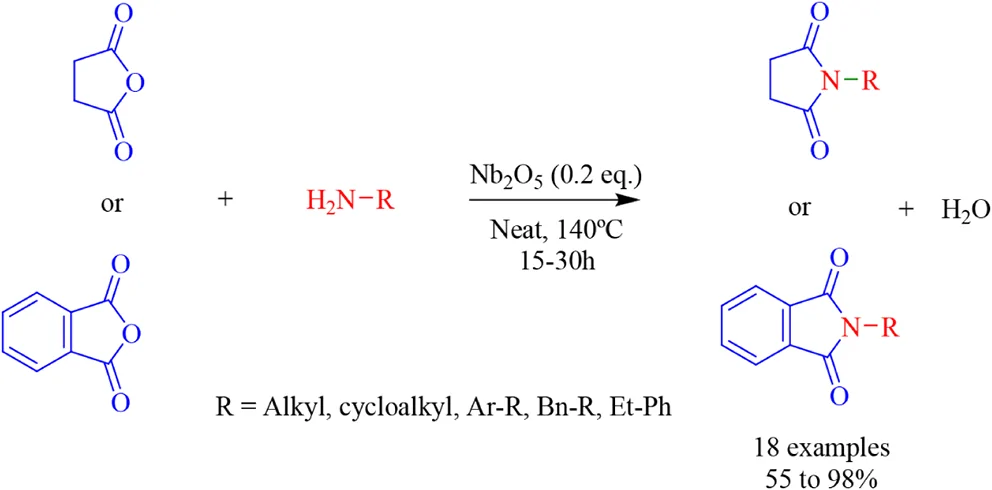
Synthesis of Cyclic Imides from Anhydrides Catalyzed by Nb_2_O_5_

Nb_2_O_5_ also promotes hydrolytic transformations, such as the hydrolyses of amides to carboxylic acids, a reaction of relevance in industrial and biochemical contexts, affording high yields (up to 97%) and broad substrate scope (Scheme 21).^10^ Comprehensive benchmarking against twenty-three acidic, basic, heterogeneous, and homogeneous catalytic systems highlighted Nb_2_O_5_ as the most efficient catalyst among those evaluated, affording 97% yield, while ZrO_2_ and CeO_2_ exhibited the closest performance, both providing around 50% yield under identical conditions. The superior activity of Nb_2_O_5_ has been associated with the synergistic contribution of Lewis acidity and moderate surface basicity, which promotes amide carbonyl activation and facilitates nucleophilic attack by water, leading to C–N bond cleavage, while maintaining catalytic performance in the presence of inhibitory species such as water and ammonia.^10^ However, these advantages come at the cost of relatively harsh conditions, requiring elevated temperatures and extended reaction times (∼20 h), highlighting a trade-off between robustness and reaction efficiency that may limit competitiveness with more recent catalytic systems.

**Scheme 21:**
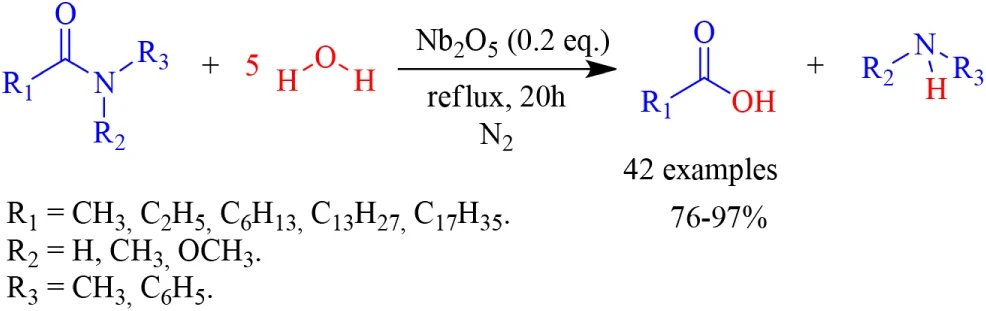
Hydrolysis of Different Aliphatic Amides to the Corresponding Carboxylic Acids Catalyzed by Nb_2_O_5_

In addition, niobium oxides have been explored in photocatalytic applications. Nb_2_O_5_ promotes C–C coupling reactions starting from acetophenone, under UV irradiation via radical pathways (Scheme 22).^50^ In these reactions the authors highlighted the formation of two main products, 2,3-diphenylbutane-2,3-diol (DPB) and 2-phenylbutane-2,3-diol (PB), in moderate yields within short irradiation times (∼2 h). Based on product analysis, the authors tentatively proposed a radical pathway wherein PB arises from cross-coupling between radicals derived from acetophenone and ethanol, which acts as the solvent.^50^ However, the requirement for a high Nb_2_O_5_ loading raises questions regarding the extent of its catalytic contribution in this reaction, as the observed activity may also involve promoter-like behavior or stoichiometric participation of the niobium oxide.

**Scheme 22:**
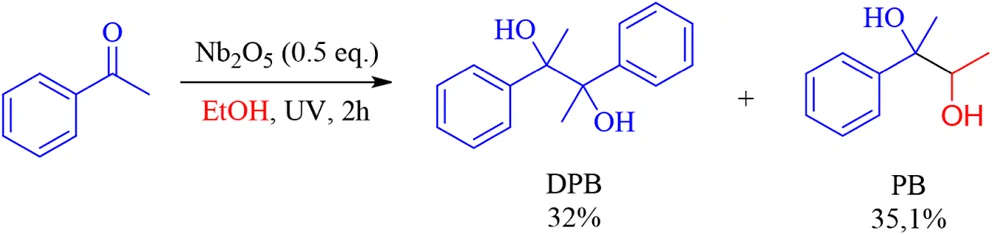
C–C Coupling from Acetophenone Photopromoted by Nb_2_O_5_

Still in the context of photocatalysis, literature reports describe the use of niobium oxide in combination with H_2_O_2_ for the photodegradation of azo dyes.^51^ The Nb_2_O_5_/H_2_O_2_ system has been applied to the degradation of methyl orange, achieving approximately 50% reduction of initial dye concentration in less than 3 h, employing 1.0 g L^–1^ of Nb_2_O_5_ and 0.20 M H_2_O_2_. Despite the short reaction times in these photocatalytic applications, the relatively high catalyst loadings and moderate yields in coupling and degradation reactions suggest that these systems still suffer from poor catalytic turnover.

In addition to the direct use of niobium oxides as catalysts in organic reactions, recent efforts have focused on the development of modified Nb_2_O_5_ materials with improved surface properties. Mesoporous and nanostructured Nb_2_O_5_ catalysts illustrate how structural control can enhance catalytic performance. For instance, mesoporous crystalline Nb_2_O_5_ (MC-Nb_2_O_5_), obtained via a sol–gel process, with high surface area (127 m^2^ g^–1^) and defined pore architecture (diameter of 5.6 nm), promoted efficient alkyne hydration to ketones in yields ranging from 22 to 99% within less than 10 h (Scheme 23).^52^ In a related approach, Nb_2_O_5_ nanoparticles prepared via supercritical CO_2_-assisted precipitation display even higher surface areas (340 m^2^ g^–1^) and promote the oxidative coupling of anilines with H_2_O_2_, affording azoxybenzene derivatives in good yields within short reaction times (∼45 min) under mild, heating-free conditions (Scheme 24).^53^ The authors compared the Nb_2_O_5_-scCO_2_-derived material with commercial oxides, including standard Nb_2_O_5_, TiO_2_–P25, and WO_3_, demonstrating its exceptional catalytic efficiency. While these reference systems consistently exhibited negligible aniline conversion (<5%) under identical mild conditions, the high-surface-area Nb_2_O_5_ material enabled rapid and selective transformation, achieving 86% conversion.

**Scheme 23:**
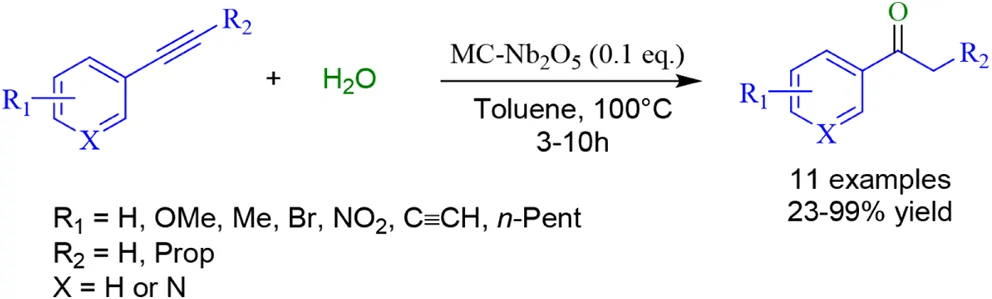
Synthesis of Ketones from Alkynes Catalyzed by Mesoporous Crystalline Nb_2_O_5_

**Scheme 24:**
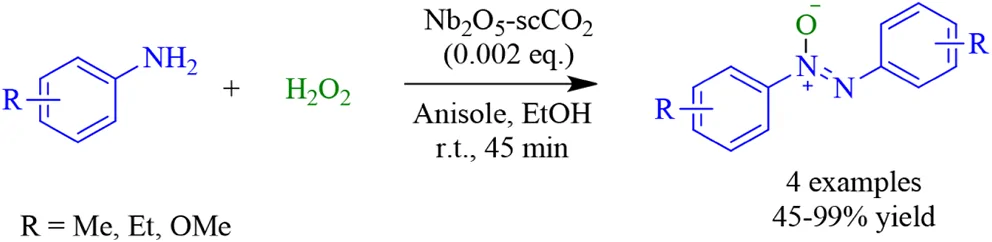
Synthesis of Azoxybenzene Catalyzed by Nb_2_O_5_ Obtained from Supercritical CO_2_-Assisted Precipitation

In both systems, the presence of Brønsted acid sites is proposed to play a key role in substrate activation and reaction promotion. The combination of low catalyst loadings, recyclability, short reaction times, and relatively mild reaction conditions renders these transformations attractive synthetic tools. Specifically, the Nb_2_O_5_-scCO_2_ system represents one of the few examples in which quantitative catalytic parameters have been reported. The calculated TON and TOF values of 129 and 172 h^–1^, respectively, based on the Nb content determined by ICP-OES, provide evidence of effective catalytic turnover and support the role of Nb_2_O_5_ as a genuine heterogeneous catalyst rather than merely a promoter. Nevertheless, while these modifications improve catalytic performance, the additional processing steps required for the preparation of modified Nb_2_O_5_ materials should be considered when evaluating their overall practical applicability and sustainability.

Similarly, niobium oxide materials (*ortho*-Nb_2_O_5_) derived from ammonium niobium oxalate provide structurally distinct catalysts with high surface area (208 m^2^ g^–1^) and broad applicability in biomass-related transformations. These systems efficiently catalyze the conversion of trioses (e.g., dihydroxyacetone, DHA) to lactic acid and the dehydration of xylose to furfural, delivering yields near 80% within ∼3 h at 120 °C (Scheme 25).^54^

**Scheme 25:**
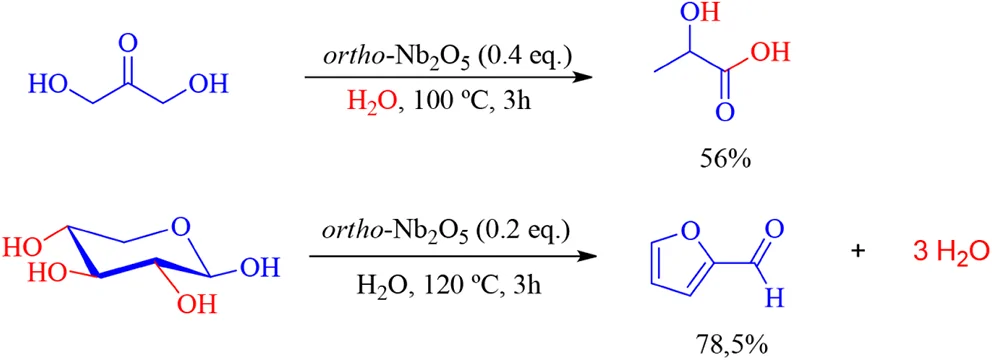
*ortho*-Nb_2_O_5_ in the Conversion of DHA and Xylose into Lactic Acid and Furfural, Respectively

Extensive comparative benchmarking against various acidic systems, including amorphous niobic acid, homogeneous Brønsted acids (H_2_SO_4_), and water-tolerant Lewis acids (Sc­(OTf)_3_), demonstrates the exceptional performance of *ortho*-Nb_2_O_5_, whose high density of water-tolerant Lewis acid sites enables efficient promotion of key reaction steps, resulting in approximately twice the yield obtained with the benchmark catalysts.

Overall, Nb_2_O_5_-based systems demonstrate broad applicability and notable robustness, particularly in transformations involving polar substrates and water-tolerant conditions. Their heterogeneous nature enables catalyst recovery and reuse, representing a clear practical advantage over NbCl_5_. In general, these systems operate with lower catalyst loadings than NbCl_5_, although they often require longer reaction times and elevated temperatures.

Importantly, advances in structural modification of Nb_2_O_5_ have led to materials with high surface areas and enhanced catalytic efficiency, enabling reactions to proceed with very low loadings (as low as ∼0.002 equiv) and short reaction times (<1 h) while maintaining excellent yields. However, the practical applicability of these advanced materials is inherently linked to their preparation, which typically involves additional reagents, multistep procedures, and, in some cases, high temperatures or specialized techniques. While catalyst recyclability partially offsets these limitations, the overall efficiency of Nb_2_O_5_-based systems ultimately depends on balancing catalyst design, preparation cost, and operational conditions.

### 3.3 Ammonium Niobium Oxalate (ANO)

Ammonium niobium oxalate (ANO) occupies an intermediate position within the niobium catalyst toolbox, combining lower toxicity, low cost, greater stability, and easier handling relative to NbCl_5_.^55^ In recent years, ANO has emerged as a versatile and comparatively greener catalyst for organic synthesis, particularly in water, glycerol, and PEG-400 media. Its most successful applications are linked to the activation of carbonyl-containing substrates, but the more relevant aspect is practical: ANO often preserves niobium-derived activation while avoiding some of the limitations associated with NbCl_5_, such as moisture sensitivity and the need for near-stoichiometric loadings.^55^

ANO efficiently catalyzes the synthesis of bis­(indolyl)­methanes through condensation of indoles with aldehydes (Scheme 26).^55^ Under aqueous conditions (0.05–0.15 equiv of ANO, 50 °C), products are obtained in good to excellent yields (65–99%) within 1–8 h. Alternatively, ultrasonic-assisted conditions in glycerol significantly reduces reaction times to minutes, albeit with more variable yields (20–99%). The system exhibits high chemoselectivity for aldehydes over ketones and allows catalyst and solvent recycling, reinforcing its compatibility with greener reaction media.^55^ An interesting comparison reported by the authors highlights the superior efficiency of ANO compared with bulk Nb_2_O_5_ in this transformation, affording yields of only 12% for Nb_2_O_5_ and approximately 97% for ANO under identical reaction conditions. This critical performance evaluation demonstrates that ANO provides a substantially more active niobium-based catalytic system, overcoming the limited reactivity typically associated with conventional niobium oxides in this specific transformation.

**Scheme 26:**
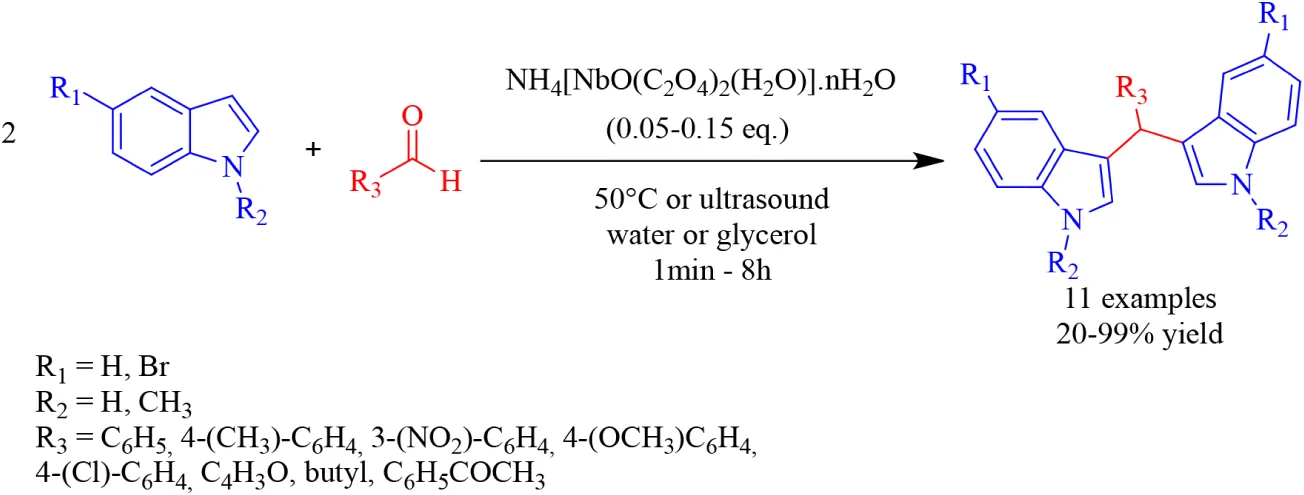
Synthesis of Bis­(indolyl)­methanes Catalyzed by ANO

ANO also promotes cyclocondensation reactions of α-phenylglyoxylic acid with *ortho*-functionalized anilines, providing benzothiazole and benzoxazinone derivatives through carbonyl activation followed by intramolecular cyclization (Scheme 27).^56^ Under optimized conditions (0.1 equiv of ANO in PEG-400 at 100 °C), moderate to excellent yields are obtained within 1–6 h, while ultrasonic irradiation further reduces reaction times to minutes without substantial loss in efficiency.^56^ Among the evaluated catalysts, ANO and Nb_2_O_5_ (0.1 equiv) afforded the desired benzothiazole in comparable yields (85% and 84%, respectively). Despite the similar catalytic performance, the authors emphasized that ANO represents a more attractive catalytic system due to its lower cost, bench stability, and easier handling compared to conventional Nb_2_O_5_, while maintaining a comparable efficiency for this transformation.

**Scheme 27:**
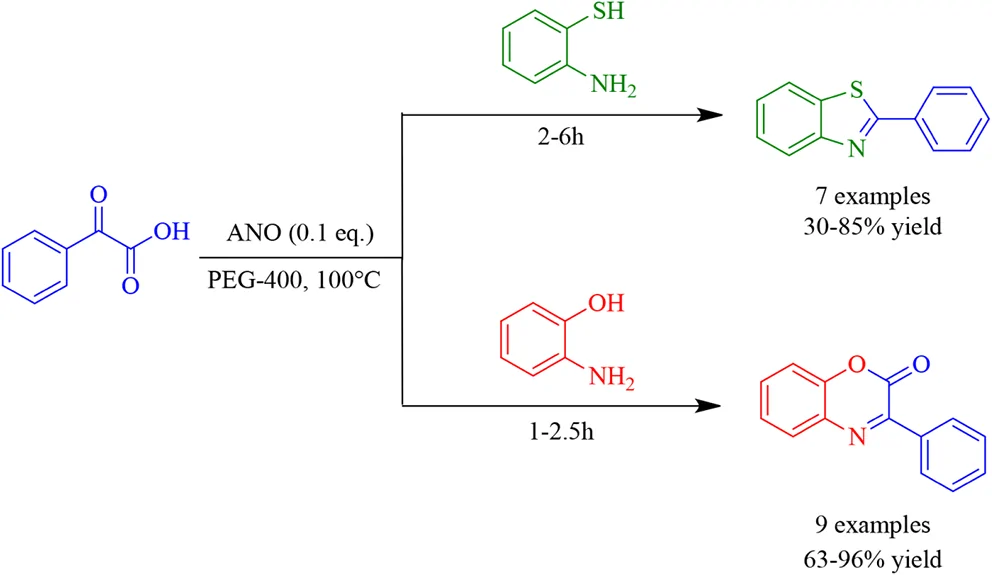
Synthesis of 2-Arylbenzothiazoles and 3-Aryl-2*H*-Benzo­[b]­[1,4]­Benzoxazin-2-Ones Catalyzed by ANO

Ultrasound-assisted ANO catalysis also enables the rapid synthesis of 3-arylquinoxalin-2­(1*H*)-ones from *o*-phenylenediamines and α-ketoacids (Scheme 28).^58^ Using PEG-400 and only 0.05 eq of the catalyst, the methodology affords yields up to 96% within 10 min and was successfully demonstrated on gram scale. Spectroscopic evidence supports the selective activation of the keto carbonyl toward Schiff-base formation and subsequent cyclization, favoring quinoxalinone formation over competing amidation pathways.^57^ Although mixtures of regioisomers may arise with unsymmetrical substrates, the methodology highlights the strong potential of ANO under process-intensified conditions.

**Scheme 28:**
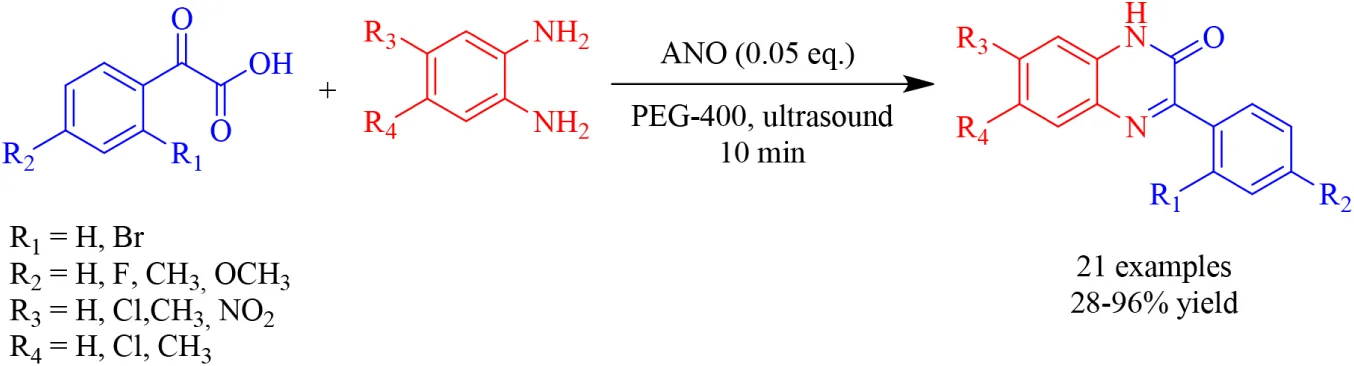
Synthesis of 3-Phenylquinoxalin-2­(1H)-One Derivatives Catalyzed by ANO

Table 2 presents a comparative overview of NbCl_5_, Nb_2_O_5_, modified Nb_2_O_5_, and ANO-based systems. Overall, ANO-mediated methodologies combine low catalyst loadings, operational simplicity, and compatibility with greener reaction media, frequently outperforming more traditional niobium sources in terms of practicality. In contrast to NbCl_5_ and several Nb_2_O_5_-based systems, many ANO-catalyzed transformations proceed under milder conditions and benefit substantially from ultrasonic irradiation, enabling significant reductions in reaction time.

**Table 2:** Comparative Summary of Representative NbCl_5_, Nb_2_O_5_- and ANO-Based Systems

System	Main transformations	Key advantages	Main limitations
NbCl_5_	Annulations, multicomponent reactions, Friedel–Crafts reactions, cycloadditions	Broad substrate scope; chemoselectivity; high yields short reaction times;	Anhydrous/inert conditions; moisture sensitivity; elevated temperatures; near-stoichiometric loadings.
Nb_2_O_5_	Amidation, hydrolysis, multicomponent reactions	Reusable; water-tolerant.	Elevated temperatures; long reaction times.
Modified Nb_2_O_5_	Hydration, oxidation, biomass conversion	Reusable; water-tolerant; high surface area; low catalyst loadings; short reaction times.	Complex catalyst preparation; possible structural instability and leaching.
ANO	Condensation and cyclization reactions	Greener media; low catalyst loadings; low cost; ultrasound compatibility.	Substrate-dependent performance.

### 3.4 Modified Niobium-Based Catalysts

Recent studies on modified niobium catalysts move the field beyond simple salts and bulk oxides. Support effects, nanostructuring, heteroatom doping, and mixed-metal compositions are increasingly employed to tune acid strength, accessibility of active sites, and resistance to deactivation, transforming niobium chemistry from a collection of isolated empirical observations into a more rationally designable catalytic platform. These modifications often improve catalyst dispersion, stability, selectivity, and overall catalytic performance relative to conventional niobium systems.^50,58–67^ To establish structure–property relationships and confirm the successful preparation of these materials, the reviewed studies employed a variety of complementary characterization techniques. Among the most common were thermogravimetric analysis (TGA) to assess thermal stability and decomposition profiles; scanning electron microscopy (SEM) to examine surface morphology and topography; X-ray diffraction (XRD) to evaluate crystallinity and phase composition; Fourier transform infrared spectroscopy (FTIR) to identify surface functional groups and chemical bonding; energy-dispersive X-ray spectroscopy (EDS) to determine elemental composition; and Brunauer–Emmett–Teller (BET) analysis to measure specific surface area and porosity, among other specialized techniques.

Supported niobium systems effectively illustrate how active-site dispersion and surface engineering dictate overall catalytic performance. For example, NbCl_5_ supported on perlite efficiently promotes multicomponent synthesis of pyran derivatives, affording high yields (up to 95%) within short reaction times (∼30 min) under relatively mild conditions (Scheme 29).^59^ The optimized 0.1 equiv loading highlights the importance of active-site dispersion rather than simply increasing catalyst quantity. Performance benchmarking against a diverse library of heterogeneous catalysts, including polyphosphoric acid-modified silicas (90%), sulfated mesoporous materials (61%), and organic catalysts (85–90%), highlights the distinct catalytic advantage of this supported system, which consistently affords higher product yields in shorter reaction times than previously reported methods. The authors further compared the supported catalyst with unsupported NbCl_5_ under analogous reaction conditions. While unsupported NbCl_5_ afforded the desired product in only 71% yield after more than 72 h, the supported catalyst achieved a 95% yield in just 15 min, clearly demonstrating the beneficial effect of catalyst immobilization on silica. Finally, the catalyst was comprehensively characterized using TGA, SEM-EDS, XRD, FTIR, and BET analyses, providing structural evidence to support its enhanced catalytic performance.

**Scheme 29:**
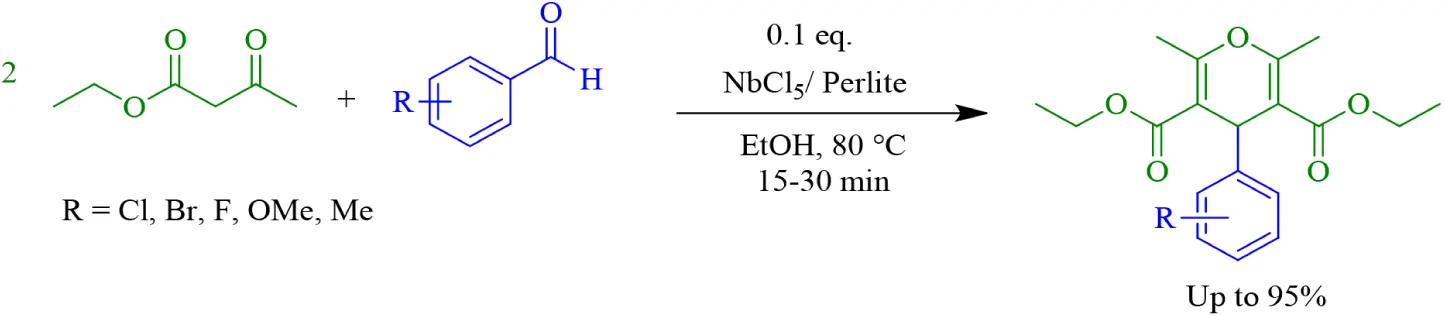
Synthesis of Pyrans Catalyzed by NbCl_5_/Perlite

In another study, the authors proposed an equimolar NbCl_5_/AgClO_4_ mixed-metal catalytic system as a more environmentally benign approach for the multicomponent synthesis of biologically important pyrazoles. Under mild and genuinely catalytic conditions, the methodology afforded the desired products in yields of up to 88% (Scheme 30). The cooperative effect between the niobium and silver species was proposed to enhance catalytic turnover while maintaining operational simplicity.^60^ The authors further compared the performance of the NbCl_5_/AgClO_4_ system with a range of previously reported catalysts, including organocatalysts such as l-proline, Zn­(l-proline)_2_, and guanidinium chloride, as well as metal salts such as CoCl_2_ and Mn­(II). Based on these comparisons, they identified the bimetallic system as a promising alternative, combining shorter reaction times and milder reaction conditions with competitive product yields. Although no physicochemical characterization of the catalytic system was reported, the authors emphasized its practical advantages, including the low catalyst loading, straightforward workup, and mild reaction conditions.

**Scheme 30:**
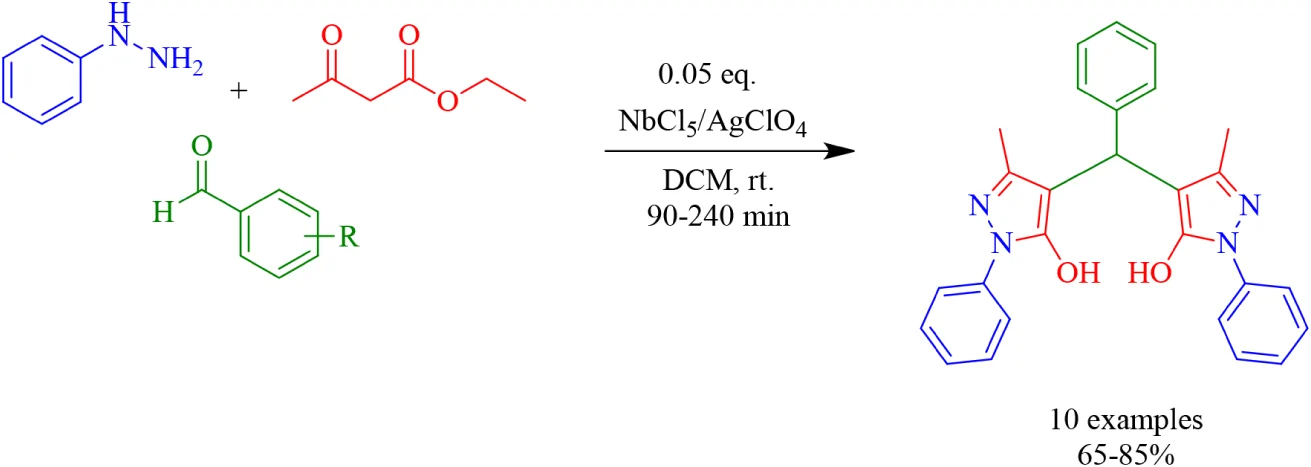
Synthesis of Pyrazoles Catalyzed by NbCl_5_/AgClO_4_

Mixed oxide systems such as Nb-WO_3_ combine Lewis and Brønsted acid sites, extending niobium catalysis beyond conventional carbonyl activation chemistry. In biomass-related transformations, these catalysts promote glucose dehydration to 5-hydroxymethylfurfural (HMF) in aqueous media, although only moderate yields (∼56%) are obtained due to the intrinsic complexity of biomass conversion processes (Scheme 31).^61^ The modified catalyst was extensively characterized by techniques such as XRD, BET, and FTIR. The authors also utilized computational models, such as DFT, to elucidate the main reaction mechanism, suggesting that the tungstite surface can adsorb glucose, abstract a proton from glucose, and catalyze the hydride shift to fructose. Nb substitution in the tungstite surface structure stabilizes the deprotonated glucose intermediate and, accordingly, lowers the overall activation barrier for glucose isomerization.

**Scheme 31:**
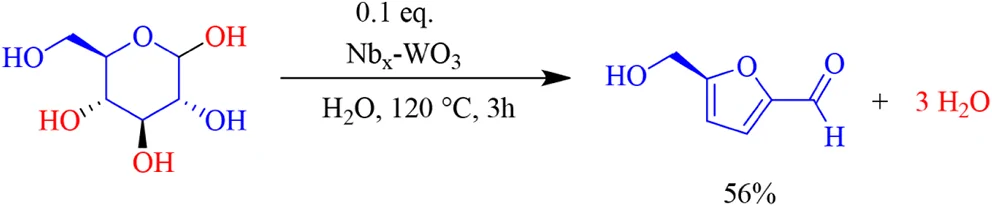
Synthesis of 5-Hydroxymethylfurfural from Glucose Using a Niobium–Tungsten Catalyst

Nb_2_O_5_-supported Pd and Ni catalysts have been applied in Suzuki–Miyaura cross-coupling reactions for the synthesis of biaryl compounds (Scheme 32).^62^ Under optimized conditions low catalyst loadings (<0.03 equiv) delivered moderate to high yields (35–91%). In this system, Nb_2_O_5_ acts as a support that promotes efficient dispersion of the active metal species. A rigorous comparison with established literature protocols highlights the advantages of this methodology. Although Pd/Nb_2_O_5_ afforded product yields (91%) comparable to those obtained with conventional Pd- and Ni-based organometallic catalysts (71–100%), it operates under milder reaction conditions, employs less toxic solvent media, and requires shorter reaction times, offering significant practical advantages over many previously reported protocols. The authors performed comprehensive physicochemical characterization with FTIR, XRD, SEM, TEM, and TGA, characterizing the catalyst structure. The catalyst can be reused for up to two additional cycles, although partial deactivation due to surface poisoning and metal leaching is observed. Despite these limitations, this study illustrates how support engineering can simultaneously enhance catalytic performance and improve the practical applicability of niobium-based catalytic systems.

**Scheme 32:**
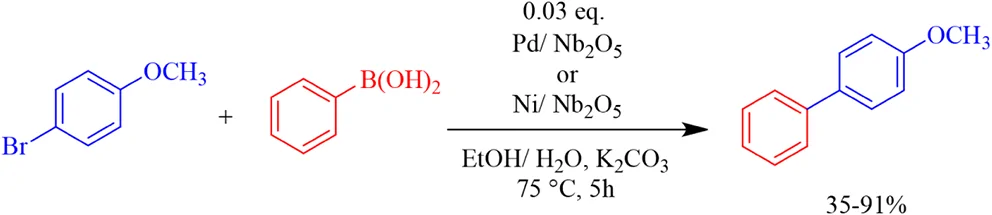
Suzuki–Miyaura Coupling Reactions Catalyzed by Pd/Nb_2_O_5_ and Ni/Nb_2_O_5_

Niobium-doped phosphomolybdic acids further illustrate how Nb incorporation can modulate acidity and catalytic performance. These Brønsted-acid systems efficiently promote condensation of β-citronellal with alcohols at room temperature, reaching quantitative conversion within 45 min (Scheme 33).^63^ Compared to previously reported vanadium-doped analogues, Nb incorporation increases the overall acidity of the system, enhancing activation of the aldehyde carbonyl group and facilitating the condensation process. However, the methodology still relies on very high catalyst loadings (1.25 equiv), limiting its efficiency despite the mild conditions and exhibiting promoter-like behavior. The authors provide a meticulous characterization of the catalysts/promoters used, carefully investigating techniques such as UV–vis absorption spectroscopy, FTIR, XRD, and SEM-EDS, in addition to potentiometric titration assays and gas chromatography–mass spectrometry. They propose a mechanism considering that either H^+^ or metal cations can activate the carbonyl group of the aldehyde. Additionally, the authors reported a turnover number (TON) of 196, a turnover frequency (TOF) of 0.11 mmol s^–1^, and a 98% yield after only 30 min using the H_4_PMo_11_Nb_1_O_40_ catalyst. These productivity metrics indicate that the material can sustain a high number of catalytic cycles while maintaining rapid substrate conversion, providing a more comprehensive assessment of catalytic performance than reaction yield alone.

**Scheme 33:**
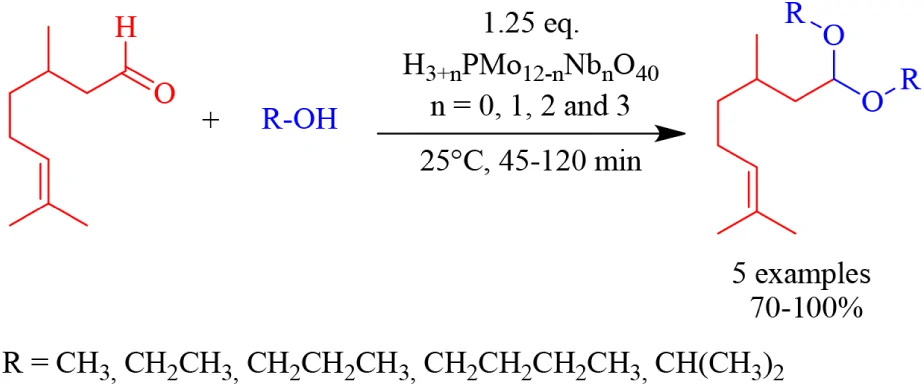
Condensation of β-Citronellal with Methanol Promoted by Niobium-Doped Phosphomolybdic Acids

Another interesting paper reported a bifunctional niobium-doped carbon nitride (Nb-CN) catalysts combining Lewis acidic Nb^5+^ centers with basic nitrogen functionalities, enabling cooperative activation in transesterification reactions (Scheme 34).^64,65^ In the synthesis of dimethyl carbonate (DMC) from ethylene carbonate (EC) and alcohols, the optimized system achieved about 54% conversion with 100% selectivity at 140 °C after 4 h using 0.045 equiv of Nb-CN.^64,65^ The authors attributed the catalytic activity to a proposed dual-activation mechanism, postulating that basic sites generate alkoxide nucleophiles while Nb^5+^ centers concurrently activate the carbonyl group toward nucleophilic attack. The catalyst also exhibits good stability and recyclability, maintaining performance over up to four reuse cycles without significant loss of activity.^65^ Comparison with state-of-the-art heterogeneous catalysts indicates that Nb-CN does not outperform the best Co-, Ce-, and Cu-based systems in terms of EC conversion (53.8% versus 70.7–84.9% under comparable conditions). Nevertheless, its quantitative selectivity toward DMC, lower susceptibility to CO_2_ poisoning, and simpler synthesis highlight a favorable balance between catalytic performance and operational practicality, making it a competitive option for applications where these attributes are more critical than achieving the highest conversion.

**Scheme 34:**
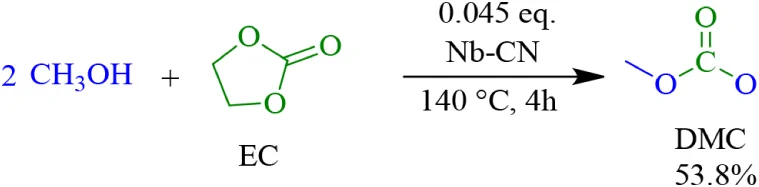
Synthesis of Dimethyl Carbonate by Transesterification of Ethylene Carbonate Catalyzed by Nb-CN

Finally, extending niobium chemistry beyond conventional Lewis acid catalysis, the NbCl_5_ system in combination with metallic magnesium provides a powerful tool for the *in situ* generation of highly reactive, low-valent Nb­(III) species. While traditional low-valent Group V systems are well-known for reducing unfunctionalized alkynes, Kadikova and coworkers demonstrated that the NbCl_5_/Mg system uniquely enables the highly regio- and stereoselective semireduction of functionalized acetylenes, specifically *N*,*N*-disubstituted 2-alkynylamines and substituted 3-alkynylols (Scheme 35). Under mild conditions, this system affords (2Z)-alkenylamines and (3Z)-alkenylols in excellent yields (73–89%).^66^

**Scheme 35:**
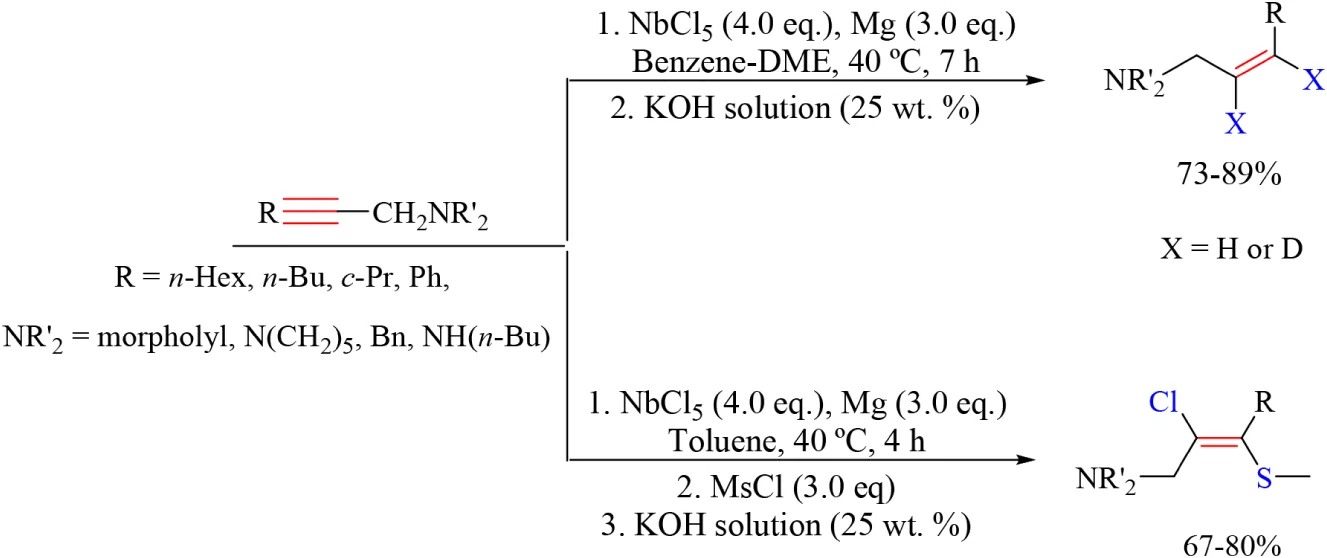
Condition-Dependent Divergent Reactivity of Substituted 2-Alkynylamines Promoted by the NbCl_5_/Mg Reagent System, Affording (Z)-Alkenylamines via Semireduction (Top) or (E)-β-Chlorovinyl Sulfides via Chlorothiolation Bottom)

Unlike many niobium-mediated processes that rely on tentatively proposed pathways, this methodology provides concrete experimental validation. Through isotopic labeling studies (deuterolysis), the authors unequivocally demonstrated the intermediacy of a stable niobiacyclopropene species. This clear experimental elucidation effectively distinguishes the proven reaction pathway from the purely speculative mechanistic interpretations often prevalent in the literature. Remarkably, this reactivity highlights a significant mechanistic divergence between niobium and tantalum: while the analogous TaCl_5_/Mg system is effective for dialkyl-substituted alkynes, it completely fails to reduce these oxygen- and nitrogen-containing functionalized substrates. This underscores the superior functional group tolerance of the low-valent niobium species. Furthermore, by switching the solvent to toluene and introducing methanesulfonyl chloride, the same NbCl_5_/Mg system can be redirected to perform regio- and stereoselective chlorothiolation of alkynes via radical pathways, yielding (E)-β-chlorovinyl sulfides.^66^

However, from a practical and sustainable perspective, this methodology currently requires superstoichiometric loadings (typically 4 equiv of NbCl_5_ and 3 equiv of Mg). While synthetically elegant, this stoichiometric bottleneck reinforces the critical need, as previously discussed, to transition these robust low-valent niobium transformations into genuinely catalytic regimes to improve their E-factors and process viability.^66^

Modified niobium-based catalysts/promoters demonstrate a clear evolution from simple Lewis acidic systems toward tunable and multifunctional catalytic platforms, where performance is strongly influenced by the local environment of Nb active sites rather than intrinsic acidity alone. Support effects, nanostructuring, and doping strategies can improve dispersion, stability, acidity, and accessibility of active sites, while cooperative Lewis/Brønsted acidity or acid–base bifunctionality enables enhanced selectivity in complex transformations. In addition, redox-active systems highlight the ability of niobium to access nonclassical reaction pathways beyond conventional Lewis acid catalysis. Despite these advances, limitations remain, including moderate efficiencies in some systems, high catalyst loadings, and incomplete mechanistic understanding, indicating that further development of structure–activity relationships is essential to fully exploit niobium in catalytic design.

## 4 Niobium in Biomass Valorization

Niobium-based catalysts have been extensively explored in biomass and biofuel conversion, where catalytic performance depends not only on product yield, but also on tolerance to oxygen-rich feedstocks, water, impurities, and repeated operation. In these systems, niobium often contributes through stabilization of acid sites, balanced Lewis/Brønsted acidity, and improved structural durability.^68–88^ At the same time, biofuel-related processes also expose some of the main limitations of niobium catalysis, since many methodologies still require elevated temperatures, long reaction times, complex catalyst formulations, or high catalyst loadings, factors that may complicate large-scale and industrial implementation.

In the field of biodiesel production, niobium catalysts have been applied in the transesterification of vegetable oils, the process of conversion of triacylglycerols into fatty acid esters (Scheme 36a).^69^ Both Nb_2_O_5_ and ammonium niobium oxalate (ANO) promote the reaction through acid-catalyzed activation of ester carbonyl groups, facilitating nucleophilic attack by alcohols. Under reflux, Nb_2_O_5_ exhibited superior activity in methanolysis reactions, achieving approximately 70% conversion after 48 h in reflux of DMSO, whereas ANO performed better in ethanolysis, reaching about 75% conversion under similar conditions.^69^ Ultrasound-assisted protocols significantly reduced reaction times to 4 h and enabled the reactions to proceed at lower temperatures, although at the expense of lower conversions (43% for ANO and 22% for Nb_2_O_5_), illustrating the trade-off between process intensification and catalytic efficiency.^69^

**Scheme 36:**
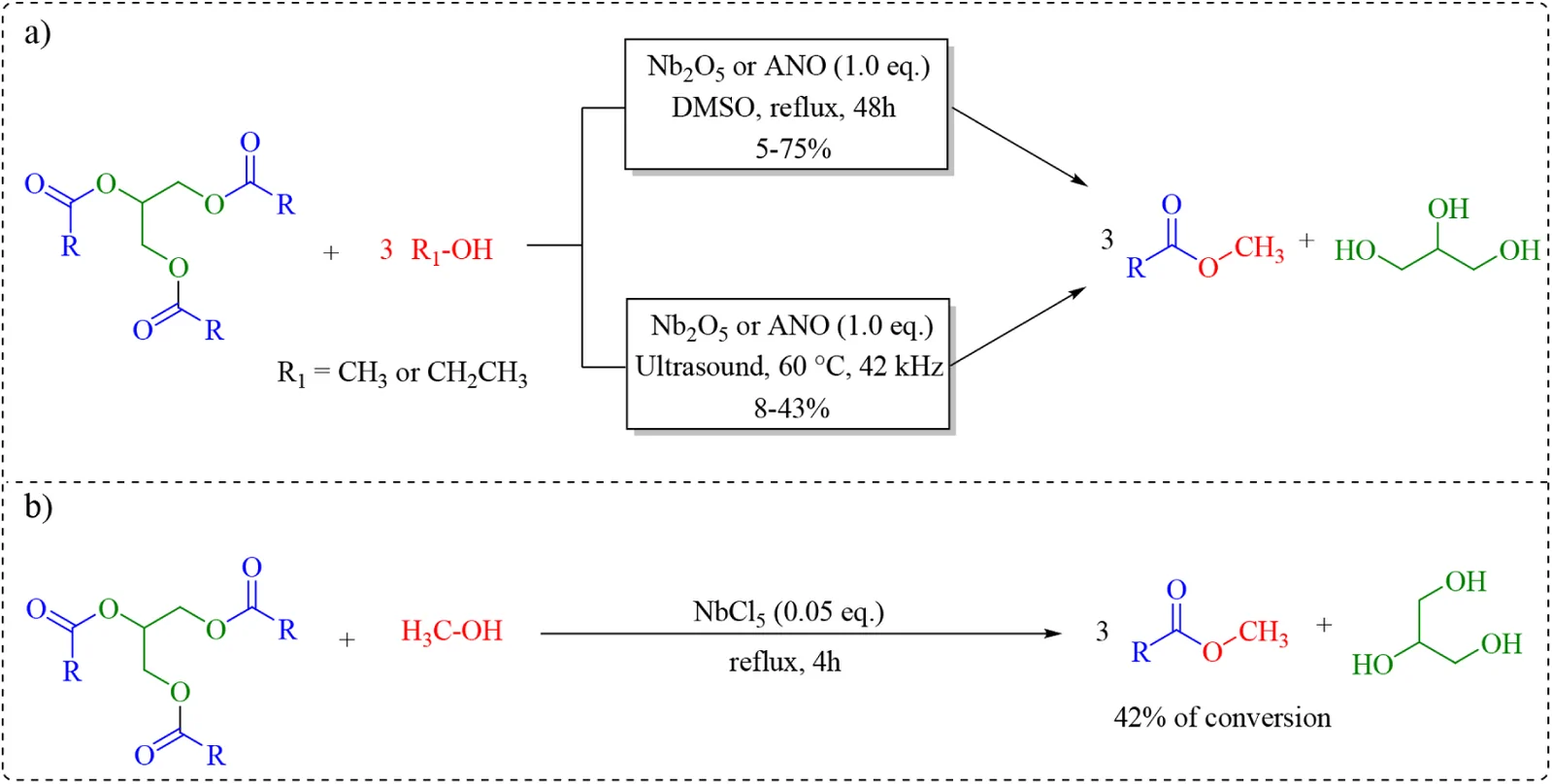
a) Transesterification of Vegetable Oils Promoted by Nb_2_O_5_ and ANO; b) Transesterification of Soybean oil Catalyzed by NbCl_5_

In a related study, the catalytic performance of NbCl_5_ was investigated in the methanolysis of soybean oil (Scheme 36b), exhibiting approximately 42% conversion under the evaluated conditions.^70^ These results underscore the critical role of strong Lewis acidity in driving transesterification, demonstrating how catalyst speciation directly governs overall reactivity. Notably, moderate conversions were achieved under simple methanol reflux using catalytic amounts of NbCl_5_ (0.05 equiv), without the need for ultrasonic irradiation. In contrast, the Nb_2_O_5_ and ANO systems required near-stoichiometric loadings (1.0 equiv), harsh thermal conditions, and, in some cases, ultrasound-assisted conditions to achieve higher conversions.^69,70^

Additional examples include niobium phosphate, sulfated niobium oxide (SO_4_^2–^/Nb_2_O_5_), and mixed CaO/Nb_2_O_5_ systems, which have have demonstrated promising activity in esterification and transesterification reactions involving oleic acid, palm oil, and macauba oil.^71–76^ Sulfation and mixed-oxide strategies generally enhance Brønsted/Lewis acidity, thermal stability, and catalyst durability, frequently leading to high ester yields and improved resistance to metal leaching during reuse cycles. In particular, (SO_4_^2–^/Nb_2_O_5_) systems achieved conversions above 90%, while mixed CaO/Nb_2_O_5_ catalysts exhibited superior stability compared to pure CaO formulations.^71–76^ Overall, these systems reinforce the central role of acidity modulation and catalyst stability in determining the efficiency of niobium-catalyzed biodiesel production.

Beyond biodiesel production, niobium-based catalysts have also attracted considerable attention in biomass valorization processes involving carbohydrates and oxygen-rich molecules.^77–89^ Biomass-derived sugars such as glucose and fructose are important renewable feedstocks to produce fuels and value-added chemicals. Among the most relevant biomass-derived platform molecules is 5-hydroxymethylfurfural (HMF), which can be converted into valuable products including FDCA, 2,5-dimethylfuran, γ-valerolactone, and levulinic acid.^11,79,80^ In these transformations, niobium-based materials are especially attractive due to their tunable acidity, thermal stability, tolerance to aqueous media, and potential for catalyst recovery and reuse.^11,71,81–84^

In this context, niobium-modified montmorillonite (Nb–Mont) has been developed as a bifunctional heterogeneous catalyst for the one-pot conversion of disaccharides into HMF (Scheme 37).^85^ Promising high yields were attained from both cellobiose (68.6% at 170 °C, 3 h, biphasic system) and sucrose (65.4% at 140 °C, 2 h, DMSO), demonstrating efficient catalytic performance in biomass valorization.^85^ The activity is attributed to synergistic Lewis/Brønsted acid sites, where Brønsted acidity promotes hydrolysis to monosaccharides, while Nb-derived Lewis sites enable glucose isomerization and subsequent dehydration to HMF. Structural analyses indicated good Nb dispersion and preservation of the montmorillonite framework, while the catalyst maintained activity over four reuse cycles with only minor deactivation.^85^

**Scheme 37:**
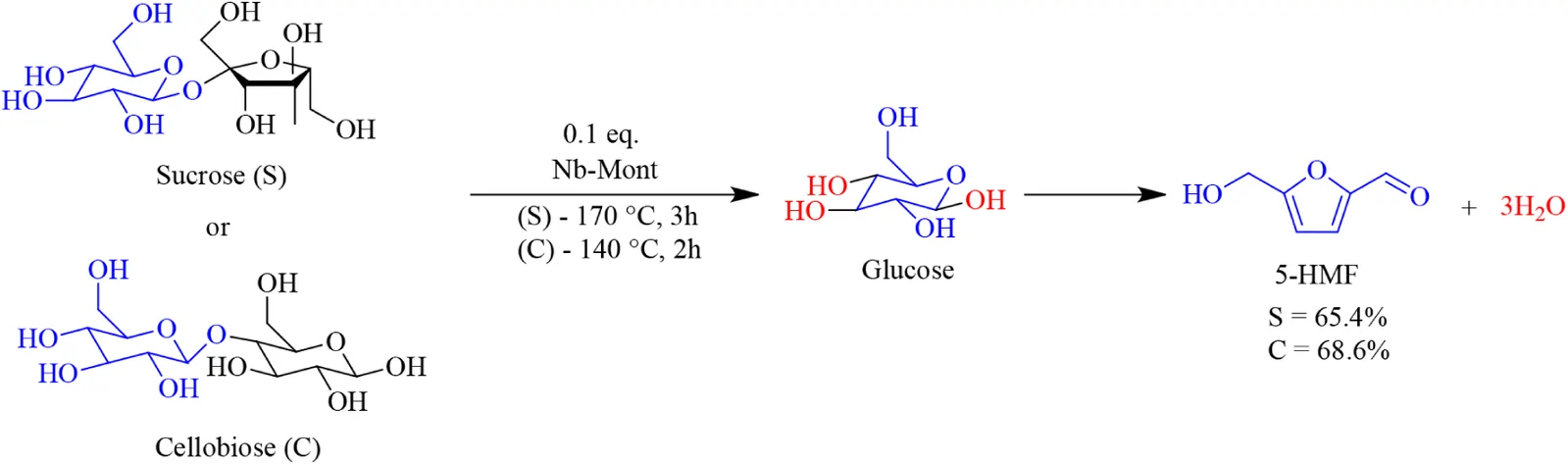
Synthesis of HMF in the Presence of Nb-Mont

Nb-doped TiO_2_ catalysts have also been applied in the conversion of cellulose to lactic acid under hydrothermal conditions (150–210 °C, 4 h, aqueous medium) (Scheme 38).^88^ The incorporation of niobium enhances the formation of water-tolerant Lewis acid sites, which are crucial for biomass transformation in aqueous environments. Catalytic performance increased with Nb content, with 0.25 equiv of Nb-TiO_2_ providing lactic acid yields of up to 29.8%, compared to only 6% for undoped TiO_2_.^88^ The improvement was attributed to enhanced Lewis acidity, facilitating key steps such as glucose isomerization and retro-aldol reactions. The catalyst also exhibited good stability over three reuse cycles.^88^

**Scheme 38:**
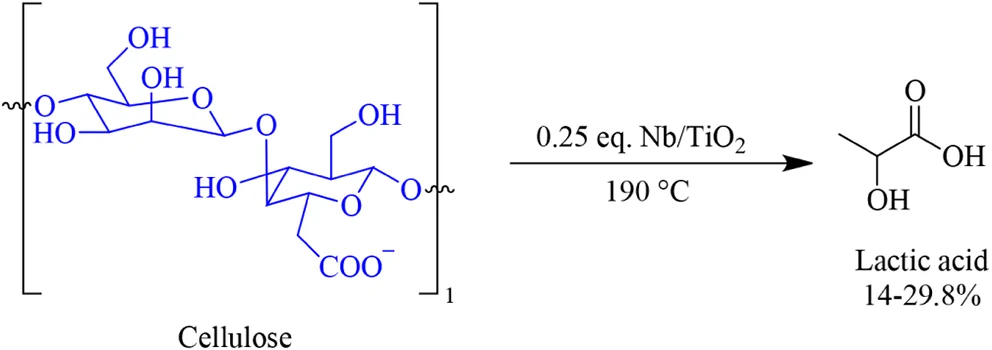
Conversion of Cellulose to Lactic Acid in the Presence of Nb/TiO_2_

Still within the context of biomass-derived sugar transformations, niobium-exchanged tungstophosphoric acid supported on TiO_2_ (NbTPA/TiO_2_) has been reported as an efficient bifunctional catalyst for the one-pot conversion of fructose to 5-ethoxymethylfurfural (EMF) via dehydrative etherification (Scheme 39).^89^ The optimized Nb_0.4_TPA/TiO_2_ catalyst achieved 99% fructose conversion and 76% EMF yield at 130 °C after 6 h, with excellent recyclability over five cycles.^89^ The high performance was attributed to the synergistic combination of Brønsted and Lewis acid sites, where Brønsted acidity promotes fructose dehydration while Nb-derived Lewis sites favor the subsequent etherification step. Excessive Brønsted acidity, however, led to side reactions such as ethyl levulinate formation, highlighting the importance of balanced acid-site distribution.^89^ Compared with several previously reported systems discussed by the authors, the NbTPA/TiO_2_ catalyst combined high conversion, good EMF selectivity, and recyclability under relatively moderate conditions.

**Scheme 39:**
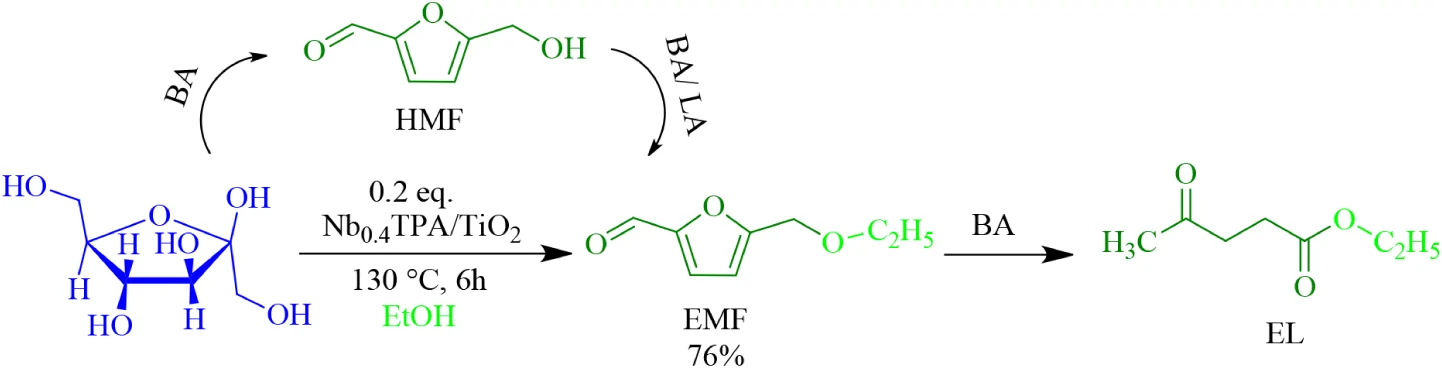
Dehydrative Etherification of Fructose to EMF Catalyzed by NbTPA/TiO_2_

In a related approach, mixed SnO_2_–Nb_2_O_5_ oxides prepared by the Pechini method exhibited promising activity in fructose conversion, with niobium incorporation significantly affecting surface area, metal dispersion, and acid–base properties.^82,90^ Compared with the pure oxides, the mixed systems exhibited a higher density of Lewis acid sites and improved catalytic performance, achieving higher fructose conversions (75–78%) while maintaining high selectivity toward HMF (60–80%).^82,90^ Overall, these studies highlight the importance of acid-site balance, catalyst dispersion, and structural stability in niobium-catalyzed biomass-derived sugar conversion. Although several systems exhibited promising selectivity and recyclability toward platform molecules such as HMF, EMF, and lactic acid, many methodologies still require elevated temperatures or carefully optimized reaction media.

Further illustrating the versatility of niobium catalysts in biomass-derived transformations, niobium-based systems have also been explored in glycerol valorization processes, including fast pyrolysis and oxidative dehydration routes.^84,91^ In fast pyrolysis, Nb-containing catalysts promoted the formation of value-added oxygenated compounds such as acids, alcohols, aldehydes, and esters, with catalytic performance strongly dependent on surface area, Nb dispersion, and acidity.^84^ Glycerol conversion increased markedly in the presence of niobium catalysts, reaching up to 82.2% for pure Nb_2_O_5_, while supported systems exhibited lower activity depending on the accessibility and dispersion of Nb active sites.^84^ In oxidative dehydration processes, mixed metal catalysts supported on Nb_2_O_5_ or niobium phosphate enabled complete glycerol conversion and improved catalyst stability due to reduced leaching and preservation of active sites during reuse cycles.^91^ Acrolein was identified as the major product, whereas acrylic acid formation remained limited, indicating that product selectivity strongly depends on catalyst composition and reaction conditions.^91^ Overall, these studies reinforce the importance of niobium not only as an acidic component, but also as a structurally stabilizing support in biomass conversion processes. Figure 3 highlights the use of niobium catalysts for biomass and biofuel conversion.

**Figure 3:**
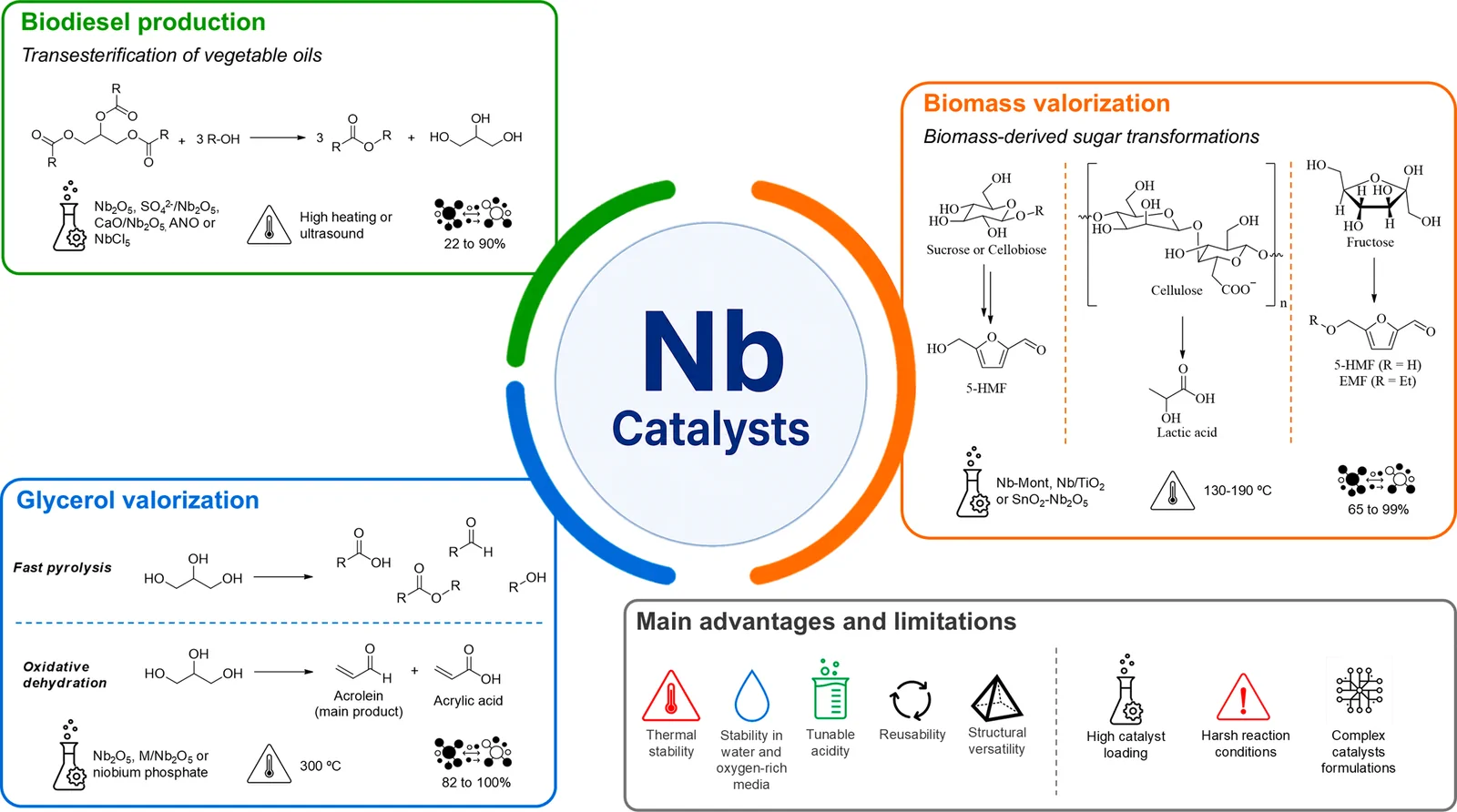
Representative applications of niobium-promoted systems in biomass valorization.

Niobium-based catalysts have demonstrated considerable versatility in biomass and biofuel conversion, particularly in reactions requiring balanced Lewis/Brønsted acidity and tolerance to oxygen-rich media. However, despite the promising catalytic performances and recyclability observed in several systems, many methodologies still depend on harsh reaction conditions, complex catalyst formulations, or carefully optimized media, which continue to limit practical large-scale application. In addition, the literature still lacks consistent process metrics, long-term stability studies, and direct techno-economic comparisons with established solid-acid catalysts, indicating that claims regarding industrial applicability should still be approached with caution.

## 5 Advantages, Limitations, and Future Perspectives

### 5.1 Comparative Assessment against Non-Niobium Catalysts

To place niobium-based systems in a broader context, Table 3 compares representative examples with non-niobium Lewis and Brønsted acid systems. Although the experimental conditions vary across reports, the comparison identifies relevant advantages and limitations without implying a direct ranking of catalytic performance.

**Table 3:** Comparative Assessment of Representative Niobium-Based Catalysts and Non-Niobium Alternatives in Selected Transformations

Reaction type	Nb-based system	Best Non-Nb Alternative	Nb advantage	Nb Disadvantage
Quinoline MCR^9^	NbCl_5_	H_2_SO_4_, Zn(OTf)_2_, FeCl_3_, and K_5_CoW_12_O_40_	Higher yields and shorter reaction time at r.t.	High loading and anhydrous conditions.
Pyrrolopyrrole MCR^29^	NbCl_5_	AcOH and TsOH	Higher yields and shorter reaction time at r.t.	High loading and anhydrous conditions.
Biginelli MCR^45^	Nb_2_O_5_	H_2_SO_4_, SbCl_3_, polyaniline-FeCl_3_, and Zn-based catalysts	Short reaction time, solvent-free conditions, and reusability.	Elevated temperature and catalyst pretreatment.
Direct Amidation^46^	Nb_2_O_5_	ZrCl_4_, Al_2_O_3_, and TiO_2_	Higher yields and reusability.	Elevated temperature and long reaction time.
Biomass conversion^54^	ortho-Nb_2_O_5_	H_2_SO_4_ and Sc(OTf)_3_	Higher yields and selectivity; water tolerance.	Elevated temperature and specific catalyst preparation.
Suzuki coupling^62^	Pd/Nb_2_O_5_	Pd- and Ni-based catalysts	Comparable yields under relatively mild conditions.	Marked deactivation and metal leaching upon reuse
Carbonate transesterification^75^	Nb-CN	Co-, Ce-, and Cu-based catalysts	Higher selectivity, reduced CO_2_ poisoning, and reusability.	Moderate conversion and elevated temperature.

The comparison demonstrates that the advantages of niobium-based systems depend strongly on the transformation and do not necessarily translate into higher product yields. For example, NbCl_5_ afforded high yields at room temperature in quinoline and pyrrolopyrrole multicomponent reactions, although high loadings and anhydrous conditions were required. Nb_2_O_5_-based systems offered complementary advantages, including solvent-free conditions, catalyst reuse, and high yields in the Biginelli and direct amidation reactions, as well as water tolerance in amidation and biomass conversion. However, achieving these benefits often required elevated temperatures, extended reaction times, and, in some cases, additional thermal or chemical steps during catalyst preparation.

The modified systems further illustrate this balance. In the Suzuki–Miyaura cross-coupling, Pd/Nb_2_O_5_ afforded yields comparable to conventional Pd- and Ni-based catalysts under relatively mild conditions but underwent marked deactivation upon reuse, with the yield decreasing from approximately 90% in the initial run to 28% in the first reuse cycle. This loss of activity was attributed to catalyst loss during filtration, minor leaching of the active phase, and poisoning by inorganic species. In another example, Nb-CN achieved complete selectivity, resistance to CO_2_ poisoning, and reusability in the production of dimethyl carbonate (DMC) through carbonate transesterification, although conversion remained moderate and an elevated reaction temperature was required.

Overall, the selection of a niobium-based system should consider the balance among activity, selectivity, operational requirements, recovery, and stability rather than product yield alone.

### 5.2 Practical, Industrial, and Sustainability Considerations

The practical relevance of niobium-based systems cannot be assessed solely from product yield or conversion. System loading, operational requirements, catalyst preparation, recovery, stability, waste generation, and scalability must also be considered. Because these parameters are reported inconsistently, a targeted screening was conducted across all 91 references included in this review. Reviews, institutional reports, conference proceedings, historical references, non-niobium benchmark studies, publications outside the scope of organic synthesis or biomass valorization, and studies focused exclusively on product characterization or subsequent applications were excluded. After applying these criteria, 61 peer-reviewed primary studies published between 2015 and 2025 remained eligible for analysis. These studies were screened for explicit evidence of recovery, reuse, leaching, deactivation, regeneration, quantitative environmental assessment, scale-up, continuous processing, residual niobium, and techno-economic analysis. Studies in which at least one of these parameters was identified were subsequently examined in full text to confirm the evidence and its experimental basis.

The practical distinction most relevant to recovery and reuse concerns the nature of the niobium system. NbCl_5_ is generally not recoverable in its original form after reaction and workup, whereas Nb_2_O_5_-based and other heterogeneous niobium materials can generally be separated from the reaction medium and evaluated for reuse. Despite this potential advantage, experimental recovery and reuse were confirmed in only 15 of the 61 eligible studies (24.6%).^17,45–47,52,54,55,59,62,65,74,75,85,88,89^ Most systems retained useful performance over the evaluated cycles. However, these experiments provide limited information on catalyst lifetime and preservation of the original active sites.

Evidence concerning leaching and deactivation was even scarcer. Experimental evaluation of leaching was identified in three studies (4.9%).^52,62,89^ Catalyst-removal tests in two studies confirmed that conversion ceased after removal of the solid, indicating no detectable contribution from catalytically active leached species under the evaluated conditions.^52,89^ In another study, postreaction X-ray fluorescence analysis suggested minor catalyst leaching after three reuse cycles.^62^ Deactivation was experimentally evaluated in only two studies (3.3%), in which activity loss was attributed to leaching, surface deposition, or partial deactivation of Lewis acid sites.^62,88^ Deactivation was also discussed, but not experimentally evaluated, in one additional study.^91^ No experimentally demonstrated catalyst regeneration was identified.

Industrial relevance is similarly difficult to establish from the available literature. Modified niobium materials may provide tuned acidity, higher surface area, improved metal dispersion, or greater recoverability, but their preparation often requires additional reagents, metals, templates, calcination, or hydrothermal treatment. These inputs are rarely considered when process advantages are discussed. Moreover, the targeted screening identified no formal techno-economic assessments or confirmed reports of residual niobium analysis in isolated products, continuous processing, or process-relevant scale-up. Consequently, important aspects of industrial feasibility, including downstream metal contamination, reactor compatibility, and process cost, remain largely unevaluated.

The reviewed literature also presents predominantly qualitative environmental claims. Solvent-free conditions, aqueous or glycerol media, short reaction times, catalyst recovery, and reuse frequently emerge as highlighted greener features.^26,44,45,53,55–57^ Although potentially advantageous, these characteristics do not establish overall environmental superiority because reaction temperature, workup, extraction, purification, and catalyst preparation may contribute substantially to material and energy consumption. Only one eligible study (1.6%) presented a quantitative environmental assessment.^76^ That study combined process simulation with the Waste Reduction Algorithm to calculate potential environmental impact per mass of product and reported a lower total impact for a heterogeneous CaO/Nb_2_O_5_ process than for a simulated homogeneous NaOH process. However, this analysis was based on a simplified process model and did not constitute a complete assessment. Established metrics such as E-factor, process mass intensity (PMI), reaction mass efficiency (RME), and solvent intensity were not identified in the evaluated reports.

Consequently, the current literature’s treatment of leaching, deactivation, and green chemistry metrics is fundamentally weak. This systemic reporting gap prevents a definitive evaluation of the true sustainability of niobium-mediated processes, underscoring an urgent need for the community to adopt standardized E-factor reporting and rigorous hot-filtration/ICP-OES leaching analyses in future studies.

Overall, the literature demonstrates the laboratory-scale potential of niobium-based systems, particularly the recoverability of heterogeneous oxides and modified materials. As summarized in Figure 4, only one-quarter of the eligible studies provided experimental evidence of recovery and reuse, whereas leaching, deactivation, regeneration, quantitative environmental performance, scale-up, and economic feasibility were addressed far less frequently. The necessarily restricted coverage of these parameters in this review therefore reflects their sparse reporting in the primary literature rather than a lack of practical importance. This reporting gap remains a central limitation of the field and constrains a more definitive assessment of the sustainability and industrial viability of niobium-based systems.

**Figure 4:**
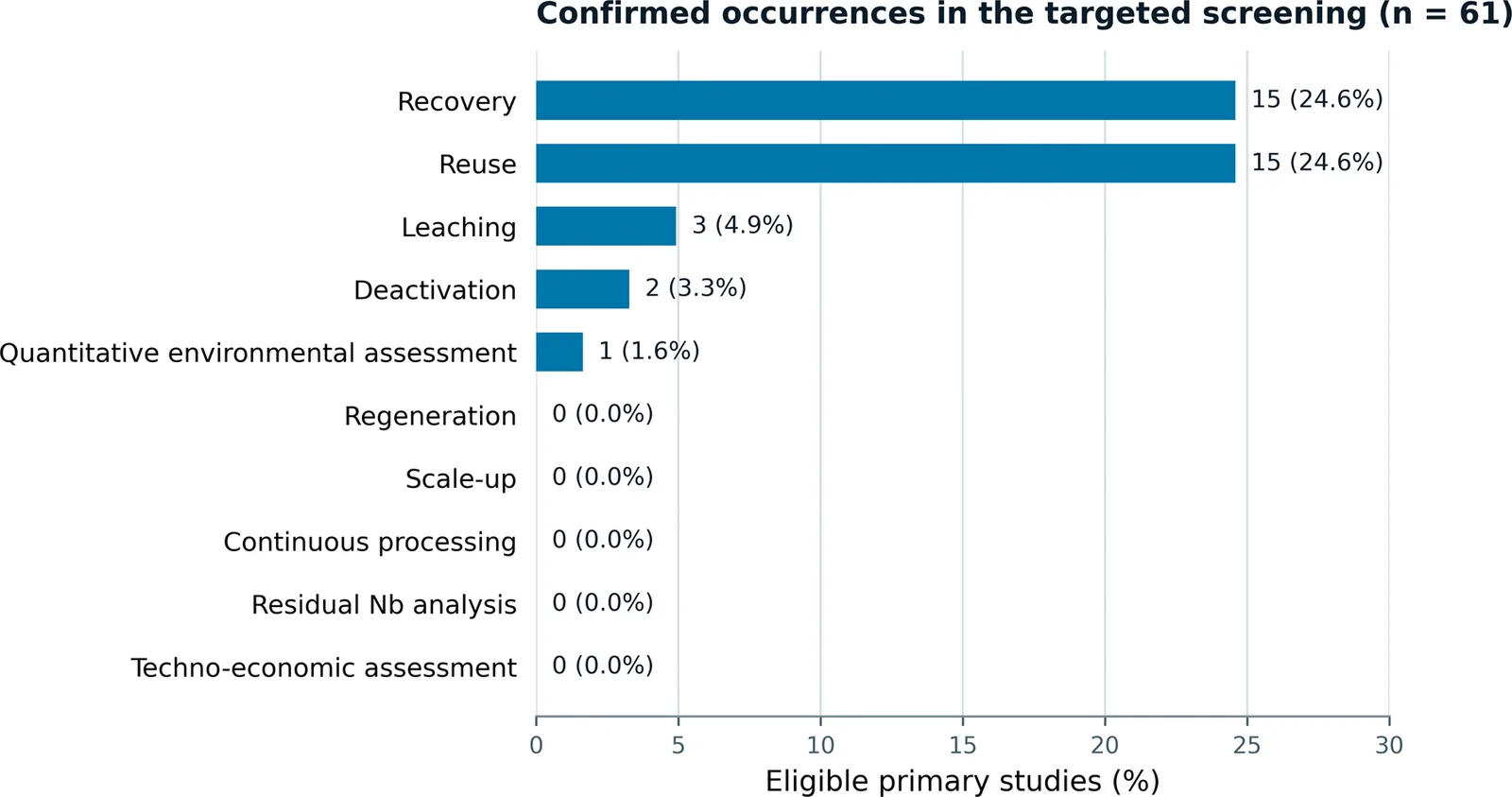
Reporting frequency of practical, industrial, and sustainability parameters among the 60 eligible primary studies.

### 5.3 Limitations and Future Perspectives

Based on the limitations identified in the preceding sections, future developments in niobium-based catalysis should focus on three critical aspects:

(i) mechanistic transparency, achieved by moving beyond empirical optimization through the integration of kinetic studies, operando or in situ spectroscopy, and computational modeling to establish more reliable structure–activity relationships and elucidate catalyst stability and deactivation mechanisms;
(ii) standardized performance and sustainability assessment, including the reporting of productivity indicators such as turnover number and turnover frequency, when applicable, as well as green chemistry metrics, energy requirements, and reuse experiments accompanied by leaching and structural stability analyses;
(iii) process-relevant validation, involving larger-scale experiments, residual-niobium analysis, continuous-flow approaches, and early stage techno-economic assessments.

Further expansion into underexplored areas, including asymmetric catalysis, electro- and photocatalytic processes, and process intensification strategies, may broaden the synthetic relevance of niobium-based systems.

Ultimately, the successful development of niobium-based catalysts will depend on the rational design of active sites, control of niobium environments, and understanding of their interaction with substrates and reaction media, enabling the selection of the most suitable niobium platform for each catalytic application.

## 6 Conclusion

This review highlights that niobium-based compounds constitute a versatile platform for modern synthesis and biomass valorization, but their true utility depends entirely on rational catalyst design rather than empirical optimization alone. By establishing strict boundaries between catalytic and promoter-like behavior, this analysis reveals that while molecular systems like NbCl_5_ frequently afford high reactivity, their broader applicability is severely constrained by superstoichiometric loading requirements and moisture sensitivity. Among the investigated classes, Nb_2_O_5_-based materials, ANO-derived systems, and rationally modified solids display the clearest practical potential, offering recoverable platforms and water-tolerant operation. Crucially, this review exposes significant systemic limitations in the current literature. Comparative benchmarking against non-niobium alternatives demonstrates that niobium is not a universally superior Lewis/Brønsted acid; its advantages in chemoselectivity and oxophilicity must be carefully weighed against operational costs and harsh conditions. Moreover, the conspicuous absence of quantitative green chemistry metrics, rigorous leaching and deactivation analyses, and industrial scalability parameters (such as continuous-flow processing and downstream contamination assessments) prevents a definitive validation of these systems for large-scale applications. Overall if niobium-based catalysis is to transition from promising laboratory-scale methodologies to viable industrial technologies, the field must move beyond isolated batch transformations. Future endeavors must prioritize operando mechanistic elucidations, standardized sustainability evaluations, and rigorous process-oriented benchmarking against established industrial catalysts.
